# Life table variations in *Wolbachia*-transinfected (*w*Mel & *w*AlbB strains) and uninfected *Aedes aegypti*: the role of various larval diets

**DOI:** 10.3389/finsc.2025.1679816

**Published:** 2025-12-12

**Authors:** Yazhini Gunasekaran, Vidhya Pachalil Thiruvoth, Sakthivel Annamalai, Vijayakumar Balakrishnan, Shriram Ananganallur Nagarajan, Manju Rahi

**Affiliations:** 1 M.Sc. Public Health Entomology, Indian Council of Medical Research (ICMR)-Vector Control Research Centre, Department of Health Research, Ministry of Health & Family Welfare, Government of India (GOI), Medical Complex, Indira Nagar, Puducherry, India; 2 Division of Vector Biology and Control, Indian Council of Medical Research (ICMR)-Vector Control Research Centre, Department of Health Research, Ministry of Health & Family Welfare, Government of India (GOI), Medical Complex, Indira Nagar, Puducherry, India; 3 Indian Council of Medical Research (ICMR)-Vector Control Research Centre, Department of Health Research, Ministry of Health & Family Welfare, Government of India (GOI), Medical Complex, Indira Nagar, Puducherry, India

**Keywords:** *Wolbachia*, larval diet, mass rearing, *Aedes aegypti*, life table, adult survival, fecundity, India

## Abstract

**Introduction:**

*Wolbachia*-based vector control strategies have been successfully implemented as a sustainable long-term solution and a promising tool for controlling *Aedes* mosquitoes, primarily *Ae. aegypti*, the main vector of major arboviral diseases. Since it is essential to rear healthy and competent adult mosquitoes for mass release under *Wolbachia*-based vector control strategies, optimising larval diet is essential. Therefore, the current study tested and compared four different larval diets to examine their statistical significance on the *Wolbachia* transinfected and uninfected *Ae. aegypti* life table traits.

**Methods:**

We tested and compared the effects of four larval diets: LD1 (fish feed), LD2 (laboratory rodent diet), LD3 (mushroom powder), and LD4 (dog biscuit plus brewer’s yeast) on hatchability, pupation, adult emergence, fecundity, and adult survival of *Wolbachia*-transinfected (*w*Mel and *w*AlbB) Puducherry strains, as Among the tested diets, fish feed (LD1) and the combination of dog biscuit with brewer’s yeast (LD4) have significant effects in both *Wolbachia*-transinfected and uninfected *Ae. aegypti* strains regarding egg hatchability, pupation, adult emergence, fecundity, and adult survival.

**Results:**

The highest fecundity was observed under LD1 for uninfected *Ae. aegypti*, with approximately 84 eggs/female (84.0 ± 6.0), followed by *w*Mel (Pud) mosquitoes (~78 eggs/female, 78.0 ± 5.2) and uninfected mosquitoes (~75 eggs/female,74.6 ± 23.3) under LD4 diet in the F0 generation. The uninfected *Ae. aegypti* females exhibited significantly lower mortality risk under LD2 (Hazard Ratio (HR)=0.56<1, P<0.001), with a high median survival of 57 days compared to all other diets.

**Discussion:**

The results of this study suggest that LD1 (fish feed) can be recommended as the superior larval diet for the mass rearing of *Wolbachia*-transinfected strains, although both LD1 and LD4 diets demonstrated positive effects on all the *Ae. aegypti* strains. Meanwhile, LD4 (dog biscuit + brewer’s yeast) can be recommended for the routine rearing of uninfected *Ae. aegypti* colonies, as it is comparatively cost-effective and readily available in India. These findings could contribute to the large-scale mosquito rearing programs under the *Wolbachia* strategy, ultimately supporting the implementation of sustainable vector control approaches for arboviral disease management.

## 1 Introduction

*Aedes aegypti* is the primary vector responsible for transmitting major arboviral diseases such as dengue and chikungunya, and it is targeted through several vector control strategies. In 2024, more than 7.6 million dengue cases have been reported worldwide, including 3.4 million confirmed cases and over 3,000 deaths (1). In India, 233,400 confirmed dengue cases and 236 deaths were reported during the same period, creating a significant public health concern that necessitates integrated approaches and effective preventive control strategies (2). Although the main prevention and control strategies for dengue have largely focused on suppressing *Aedes* populations, their effectiveness in reducing disease incidence and outbreaks remains limited in many endemic countries due to multiple factors.

*Wolbachia* is a gram-negative symbiotic bacterium that occurs naturally in around 60% of insect species and has been detected in 39.5% of the 147 mosquito species studied (3). The *Wolbachia pipientis* (*w*Pip) strain was originally identified in *Culex pipiens* mosquitoes by Hertig and Wolbach in 1924. (4). Other mosquito species harboring *Wolbachia* include *Culex quinquefasciatus, Aedes fluviatilis*, and *Aedes albopictus* (5). At the same time, *Wolbachia* has been detected at low frequency and density in *Ae. aegypti*, this remains inconclusive (6)*. Wolbachia* manipulates arthropod reproduction through four primary mechanisms: feminization of males, cytoplasmic incompatibility, selective male killing, and parthenogenesis. Additionally, *Wolbachia* interferes with arboviral replication by competing for cellular resources like cholesterol, pre-activating the mosquito immune system, triggering the phenol-oxidase pathway, and modulating miRNA-mediated immune cascades (7).

Certain *Wolbachia* strains impose fitness costs on their mosquito hosts, potentially affecting their establishment in wild populations. The highly pathogenic *w*MelPop strain markedly decreases egg viability, shortens adult lifespan, and lowers reproductive capacity, while such impacts are either milder or not observed in the *w*Mel and *w*AlbB strains. Laboratory studies have demonstrated that trans-infection of *Ae. aegypti* with *w*MelPop*, w*Mel, and *w*AlbB significantly reduce vector competence for dengue virus (8). In limited field trials, *Wolbachia* has been effectively introduced into local *Ae. aegypti* populations, where it has inhibited dengue virus replication (9). *Ae. aegypti* mosquitoes infected with *Wolbachia* can be employed for either population suppression or population replacement strategies, both of which exploit Cytoplasmic Incompatibility (CI) (10).

In the population replacement strategy, the infected females are advantaged over uninfected females because they successfully mate with both infected and uninfected males, while wild females are sterilized by the infected males. Therefore, the infection is expected to spread into the population gradually, leading to its replacement with a population incapable of transmitting viruses (11, 12). In contrast, population suppression involves releasing only *Wolbachia*-infected males, producing inviable offspring when mating with wild females. This method requires continuous releases to maintain suppression and is most effective in isolated or controlled environments where re-infestation can be minimized (13, 14). Suppression-based field trials have yielded significant reductions in dengue incidence in various regions, including French Polynesia (15), the USA (16), China (17), Singapore (13, 18), Mexico (19), and Brazil (20).

The population suppression strategy uses both Sterile Insect Technique (SIT) and Incompatible Insect Technique (IIT) in the field for dengue vector control. The SIT induces severe mutations in the male germ line, leading to defective and infertile sperm, while IIT represents only a conditional form of sterility since males remain fully fertile when mating with females harboring the same *Wolbachia* strain (21). IIT is a population suppression strategy based on exploiting CI that occurs when infected males mate with uninfected females or with females harboring a different and incompatible *Wolbachia* strain (9). Several field trials have demonstrated the potential of both SIT and IIT in reducing vector populations and dengue incidence in intervention areas (22 & 18, 20).

The implementation of *Wolbachia*-transinfected *Ae. aegypti* in field programs require the mass rearing of large numbers of healthy and competitive adults. The optimization of larval diet is a crucial component in mass-rearing programs, as proper nutrition enhances survival rates, developmental efficiency, flight capability, and mating competitiveness, ensuring uniform mosquito quality for release. Adult longevity, a component of vectorial capacity, is a proxy for vector competence (19, 23). Optimal larval development requires sufficient reserves of proteins, glycogen, amino acids, and fatty acids (24), and nutritional deficiencies may lead to higher immature mortality, weaker adults, and reduced reproductive success (25–27).

Previous studies evaluated different larval diets for their effect on *Ae. aegypti* fitness. In Mexico, protein-based diets such as tilapia fish food, bovine liver powder, and porcine meal positively affected *Wolbachia*-infected *Ae. aegypti* development (28). In Sri Lanka, dry fish powder combined with brewer’s yeast promoted better larval growth, higher fecundity, and increased male longevity compared to the International Atomic Energy Agency- (IAEA) recommended diet (29). Similarly, (21) reported that laboratory rodent diets improved adult body size and fecundity due to their higher carbohydrate content. Substituting animal proteins with plant proteins in larval diets has been shown to decrease hatchability, prolong larval development, and reduce reproductive rates (30). In collaboration with the World Mosquito Program at Monash University, Australia, the Indian Council of Medical Research-Vector Control Research Centre (ICMR-VCRC) developed two *Wolbachia*-infected *Ae. aegypti* strains native to Puducherry (referred to as Pud strains). This was achieved by backcrossing wild-type Puducherry males with Australian females carrying the *w*Mel and *w*AlbB *Wolbachia* strains. These newly established strains were assessed for biological fitness, *Wolbachia* stability, and sensitivity to insecticides (31). The findings indicated that both *w*Mel and *w*AlbB infections enhanced traits such as wing size, reproductive output, egg viability, and adult longevity when compared to the uninfected population.

Based on these findings, the present study was designed to evaluate the effects of various larval diets on the life table traits of *Wolbachia*-transinfected *w*Mel (Pud) and *w*AlbB (Pud) strains compared to uninfected *Ae. aegypti* laboratory colonies. Therefore, this study compared the standard diet with alternative diets, including fish feed, laboratory rodent diet, and mushroom powder, to assess their impact on life table characteristics. It was hypothesized that these larval diets would significantly influence developmental and reproductive traits and could be optimized for mass rearing of *Wolbachia*-transinfected mosquitoes. The study evaluated these effects across two generations to assess how larval nutrition may influence adult traits and be inherited by progeny. Life table characteristics such as hatchability, pupation rate, adult emergence, adult Male-Female ratio, fecundity, and adult survival were assessed and compared across F0 and F1 generations.

## 2 Materials and methods

**Figure 1:**
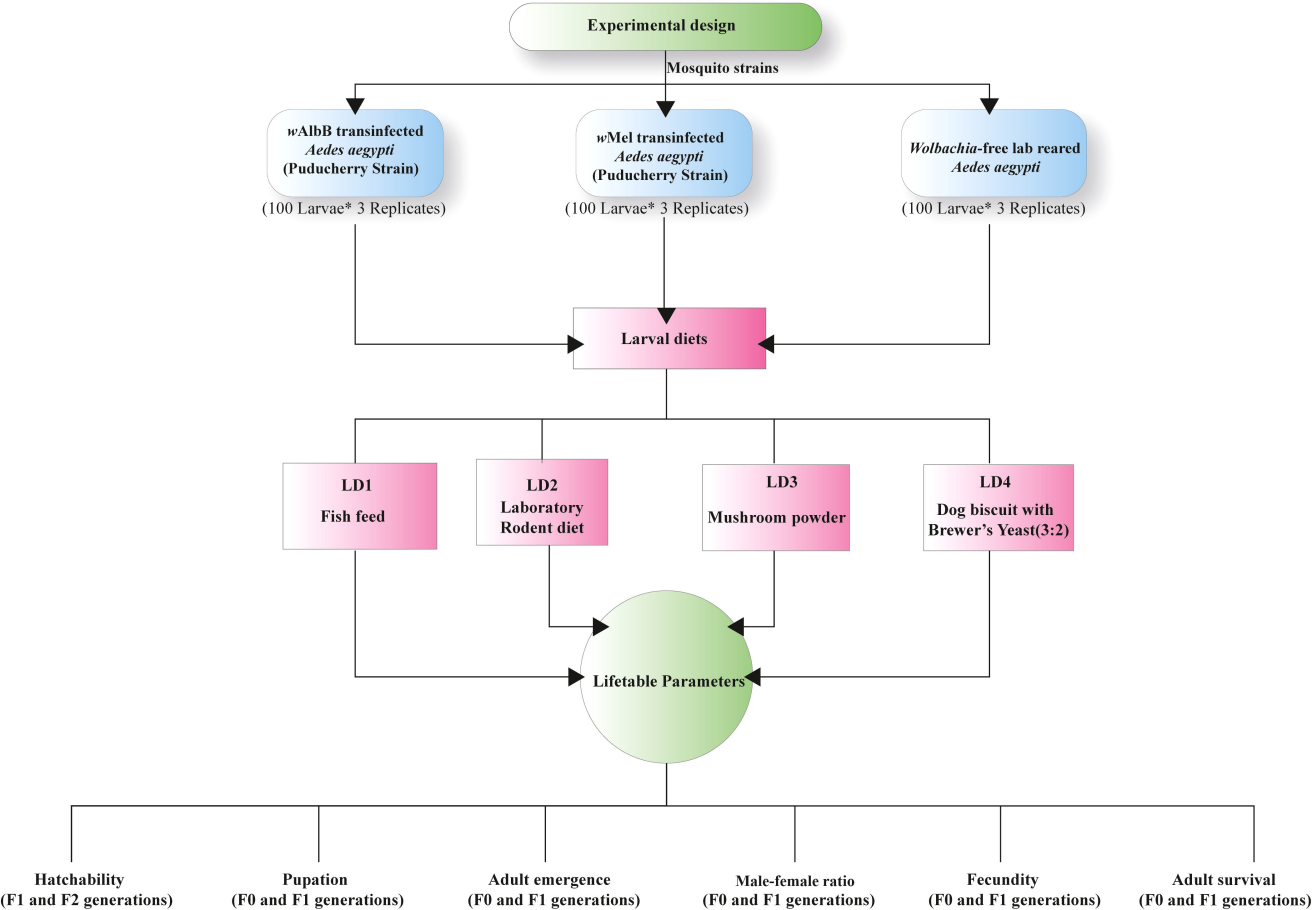
Experimental design for evaluating the effects of four larval diets on life table parameters in three *Aedes aegypti* strains: *w*AlbB (Pud), *w*Mel (Pud), and *Wolbachia*-free (lab-reared, Puducherry strain). Each experimental group consisted of 100 larvae per replicate, with three replicates per treatment (n = 300 larvae per mosquito strain per diet).

The study was conducted at the ICMR-VCRC, Puducherry, from May 2024 to August 2024. The effects of different larval diets, such as laboratory rodent diet, mushroom powder (*Agaricus bisporus*), fish feed (TetraBits), and a combination of dog biscuit with brewer’s yeast (3:2), were evaluated on *Wolbachia* transinfected *Ae. aegypti* (*w*Mel (Pud) & *w*AlbB (Pud) strains) and laboratory-reared uninfected *Ae. aegypti* mosquitoes. Life table parameters such as hatchability, pupation rate, adult emergence, and adult survival were assessed for mosquitoes reared on each diet (**Figure 1**).

### 2.1 Larval diets

Although recommended larval diets for mass rearing have been established based on previous research, the availability of easily accessible and cost-effective larval diets remains important in the Indian context. Therefore, four commercially available larval diets were selected for the present study: (1) Fish feed (Tetra, Germany), (2) Laboratory rodent diet (VRK Nutritional solutions, Pune, Maharashtra, India), (3) Mushroom powder (Transdelta Agro Products, Maharashtra, India) and (4) Dog biscuit (60%) with brewer’s yeast (40%) (in the ratio of 3:2). Additionally, fish feed has shown promising larval growth for *Wolbachia*-transinfected mosquito rearing at the ICMR-VCRC insectary.

Dog biscuits serve as an excellent source of proteins, animal fat, calcium, and vitamins (32), while brewer’s yeast contains high levels of proteins, starch, sugar, minerals, and vitamins (**Supplementary Table S1**). The mushroom powder, derived from 100% natural button mushroom (*A. bisporus*), offers an alternative plant-based protein source and contains both high proteins and carbohydrates, which are essential for energy storage, utilization, and larval growth (29, 30, 33).

For convenience, the diets were coded as follows: LD1 for fish feed (TetraBits), LD2 for laboratory rodent diet, LD3 for mushroom powder, and LD4 for dog biscuit with brewer’s yeast LD1 granules and LD2 pellets were dried and ground into powder form before being fed to the larvae. Each diet was stored in a separate clean container. The larval feeding dosage was assessed based on larval age, following the method described by Jeffrey Gutiérrez et al. (34), and food was provided on alternate days. All diet experiments were initiated simultaneously, with three replicates for each mosquito strain under each diet regimen (4 diets × 3 strains × 3 replicates). The trays were regularly monitored to assess water loss due to evaporation and larval food intake.

### 2.2 Laboratory colonization of the mosquitoes

First instar larvae of *w*Mel (Pud) and *w*AlbB (Pud) strains of *Ae. aegypti* (Pud) and uninfected laboratory-reared *Ae. aegypti* were obtained from the routine cyclical colony maintained at ICMR-VCRC. The experimental design employed three biological replicates of 100 larvae (n=300) for each mosquito strain and diet regimen. This sample size meets WHO guidelines for insect bioassays, which recommend 25–100 individuals per experiment (35). First instar larvae were transferred within 24 hours of hatching to labeled enamel trays (45 cm L × 30 cm W × 5 cm H), each containing 3 L of water, following World Mosquito Program protocols (36). Mosquito colonies were maintained at 27 ± 2 °C temperature and 80 ± 10% Relative Humidity (RH) under a 12-hour photoperiod. All replicates containing pupae were transferred to Bugdorm cages (30cm × 30cm × 30 cm) for adult emergence. Adult mosquitoes were maintained at a 10% sucrose solution, and after five days, female mosquitoes received chicken blood meals using the wax pot-collagen membrane method (36). Three days post-blood feeding, oviposition cups (200 mL capacity) partially filled with tap water were introduced for two days. Egg-laid papers were collected on the third day, air-dried at ambient conditions (27 ± 1 °C temperature, 80 ± 10% RH), and stored in sealed containers with saturated potassium chloride solution. The identical rearing procedure was repeated for the F1 generation, maintaining the same experimental design.

### 2.3 Life table analysis

A life table is a dataset that describes mortality rates across various age groups within a population of organisms (37). The present study focused on life table traits such as hatchability, pupation rate, adult emergence rate, fecundity rate, and survivorship of *Wolbachia*-transinfected *w*AlbB (Pud) and *w*Mel (Pud) strains of *Ae. aegypti*, in comparison to *Wolbachia*-free laboratory-reared *Ae. aegypti* mosquitoes. Developmental progression from eggs to larvae, larvae to pupae, and pupae to adults was observed daily and recorded for each larval diet group. The hatchability of the F0 generation was not considered in the analysis, as the study was initiated with first instar larvae. The first generation of adults was designated as the F0 generation, and the eggs they laid were considered the next generation. Therefore, the first instar larvae hatched from each generation were used to continue the subsequent generation experiment.

The pupation rate was defined as the proportion of larvae that successfully pupated, while hatchability referred to the percentage of eggs that hatched into larvae. Daily mortality was recorded for each developmental stage under each diet regimen. For fecundity, the total number of eggs laid in all oviposition cups was counted, and the average number of eggs laid per female per gonotrophic cycle was calculated.

The adult survival rate of *w*Mel (Pud), *w*AlbB *Ae. aegypti* (Pud) strains and uninfected *Ae. aegypti* strains were analyzed using a separate cohort of 100 adult mosquitoes (50 males: 50 females) per replicate for each strain and diet. To ensure equal numbers of both sexes, a separate cohort of pupae reared under the same dietary conditions was utilized to initiate the experiment. A 10% sucrose solution was provided weekly, and females were blood-fed using chicken blood. Three days post-blood feeding, oviposition cups were placed inside the cages for egg laying. Mortality of both males and females was recorded daily until all the adults died. The entire procedure was repeated for the next generation.

### 2.4 Data analysis

Data for hatchability, pupal formation, adult emergence and fecundity rates were expressed as mean ± standard deviation for each generation separately. Mixed-effects regression models were applied to evaluate differences among diets for each outcome variable, considering the hierarchical structure of the experiment. Generation (two levels) and replicate were included as random effects to account for between-generation and between-replicate variability, while diet was treated as a fixed effect. This modeling approach ensured accurate estimation of diet effects while accounting for the non-independence of observations. The Shapiro wilk test was used to assess whether the data follow a normal distribution or not. A p-value less than 0.05 was considered statistically significant. The mixed model-regression analysis results are detailed in the supplementary file no.2. Kaplan–Meier survival curves were generated for males and females of each mosquito strain, and the log-rank test was used to compare the median survival times (50%) among strains. To assess mortality risk, a Cox proportional hazards model was employed, providing hazard ratios (HR) and 95% confidence intervals separately for males and females. All statistical analyses were conducted using STATA version 14.2 (Texas, USA) and Microsoft Excel.

## 3 Results

### 3.1 Life table analysis

#### 3.1.1 Hatchability

Based on the hatchability data, both the LD1 and LD4 diets had significant positive effects on all three *Ae. aegypti* lines. For the uninfected strain, hatchability was significantly higher on LD1. For the *w*AlbB strain, hatchability was significantly lower on LD2 and LD3 compared to LD1,but showed no significant difference on LD4. For the *w*Mel strain, hatchability was significantly higher on LD4.

In the F1 generation, the hatchability ranged from 80.0% to 95.0% across the four diets for uninfected *Ae. aegypti* (**Table 1**). For the *w*AlbB (Pud) strain, hatchability was highest in LD4 (92.3%) and LD1 (89.7%), while for the *w*Mel (Pud) strain, it was similarly highest in LD4 (87.7%) and LD1 (78.3%). In the F2 generation, hatchability ranged from 77.0% (LD2) to 94.3% (LD1) in uninfected *Ae. aegypti*, from 71.3% (LD3) to 86.3% (LD1) in the *w*AlbB (Pud) strain, and from 28.0% (LD2) to 84.7% (LD4) in the *w*Mel (Pud) strain.

**Table 1:** Life table traits of the three *Ae. aegypti* strains under four different diets in both F0 and F1 generations.

Life table traits	Uninfected *Ae. aegypti* strain			
	F0 mean (SD)	F1 mean (SD)	Beta (SE)	P-value
Hatchability (F1 & F2)				
LD1	95.00 (3.00)	94.33 (3.05)	Ref	–
LD2	80.00 (8.54)	77.00 (8.18)	-16.17 (2.64)	<0.001
LD3	91.00 (3.61)	86.67 (4.16)	-5.83 (2.64)	0.027
LD4	91.33 (4.04)	86.33 (4.04)	-5.83 (2.64)	0.027
Pupation rate				
LD1	99.00 (1.00)	97.33 (1.53)	Ref	–
LD2	96.33 (4.73)	90.00 (1.00)	-5.00 (1.89)	0.008
LD3	96.33 (0.58)	85.00 (3.00)	-7.50 (1.89)	<0.001
LD4	95.67 (4.16)	96.00 (2.00)	-2.33 (1.89)	0.218
Adult emergence				
LD1	99.00 (1.00)	95.33 (2.08)	Ref	–
LD2	96.33 (4.73)	90.00 (1.00)	-4.00 (1.85)	0.031
LD3	96.33 (0.58)	85.00 (3.00)	-6.50 (1.85)	<0.001
LD4	95.67 (4.16)	96.00 (2.00)	-1.33 (1.85)	0.472
Female sex %				
LD1	49.50 (0.50)	48.95 (0.90)	Ref	–
LD2	47.76 (3.04)	48.52 (0.84)	-1.09 (0.63)	0.084
LD3	48.44 (0.52)	48.25 (0.53)	-0.88 (0.63)	0.160
LD4	48.77 (0.63)	48.28 (1.16)	-0.70 (0.63)	0.262
Fecundity				
LD1	83.91 (5.94)	45.57 (9.45)	Ref	–
LD2	71.37 (14.78)	75.03 (7.16)	8.46 (11.11)	0.446
LD3	68.83 (23.29)	9.63 (2.96)	-25.50 (11.11)	0.022
LD4	74.67 (23.38)	86.43 (1.94)	15.81 (11.11)	0.155
*w*AlbB (Pud) *Ae. aegypti* strain				
Hatchability (F1 & F2)				
LD1	89.67 (3.05)	86.33 (5.13)	Ref	–
LD2	76.67 (2.08)	72.67 (2.08)	13.33 (1.94)	<0.001
LD3	73.67 (8.50)	71.33 (9.07)	15.50 (1.94)	<0.001
LD4	92.33 (3.51)	88.67 (4.04)	2.50 (1.94)	0.197
Pupation rate				
LD1	97.33 (1.53)	99.00 (1.00)	Ref	–
LD2	97.00 (1.00)	85.00 (2.65)	-7.17 (2.11)	0.001
LD3	86.33 (1.53)	90.67 (4.16)	-9.67 (2.11)	<0.001
LD4	96.00 (1.00)	97.67 (1.53)	-1.33 (2.11)	0.528
Adult emergence				
LD1	97.33 (1.53)	98.00 (1.00)	Ref	–
LD2	96.33 (0.58)	85.00 (2.65)	-7.00 (2.02)	0.001
LD3	86.00 (1.00)	90.67 (4.16)	-9.33 (2.02)	<0.001
LD4	96.00 (1.00)	97.67 (1.15)	-0.83 (2.02)	0.679
Female sex %				
LD1	49.66 (0.29)	47.29 (2.48)	Ref	–
LD2	47.74 (1.83)	47.86 (0.84)	-0.67 (1.04)	0.517
LD3	46.49 (3.59)	46.29 (1.38)	-2.09 (1.04)	0.045
LD4	48.26 (0.30)	45.06 (2.13)	-1.82 (1.04)	0.081
Fecundity				
LD1	46.92 (9.58)	60.10 (21.62)	Ref	–
LD2	61.77 (27.44)	42.97 (3.71)	-1.15 (9.40)	0.903
LD3	35.23 (11.31)	53.33 (5.02)	-9.23 (9.40)	0.326
LD4	41.97 (23.26)	71.93 (8.80)	3.44 (9.40)	0.715
*w*Mel (Pud) *Ae. aegypti* strain				
Hatchability (F1 & F2)				
LD1	78.33 (4.62)	78.33 (2.08)	Ref	–
LD2	31.33 (2.52)	28.00 (2.00)	-48.67 (1.48)	<0.001
LD3	45.00 (3.00)	42.33 (2.52)	-34.67 (1.48)	<0.001
LD4	87.67 (3.06)	84.67 (2.52)	7.83 (1.48)	<0.001
Pupation rate				
LD1	94.67 (2.08)	98.67 (0.58)	Ref	–
LD2	98.33 (0.58)	78.00 (5.57)	-8.50 (3.23)	0.009
LD3	94.33 (2.08)	96.33 (1.15)	-1.33 (3.23)	0.680
LD4	96.67 (2.08)	99.33 (0.58)	1.33 (3.23)	0.680
Adult emergence				
LD1	94.67 (2.08)	96.33 (2.52)	Ref	–
LD2	97.00 (1.73)	78.00 (5.57)	-8.00 (3.08)	0.009
LD3	94.33 (2.08)	96.33 (1.15)	-0.17 (3.08)	0.957
LD4	96.67 (2.08)	94.33 (6.02)	0.00 (3.08)	0.999
Female sex %				
LD1	48.95 (0.50)	48.46 (0.85)	Ref	–
LD2	49.14 (0.31)	47.36 (1.90)	-0.46 (0.50)	0.356
LD3	48.41 (0.90)	48.44 (1.06)	-0.28 (0.50)	0.573
LD4	48.62 (0.29)	47.78 (2.43)	-0.50 (0.50)	0.310
Fecundity				
LD1	71.12 (9.15)	45.73 (13.50)	Ref	–
LD2	65.83 (17.19)	51.43 (5.75)	0.21 (6.89)	0.976
LD3	72.70 (6.72)	26.03 (3.07)	-9.06 (6.89)	0.189
LD4	77.97 (5.30)	24.53 (12.00)	-7.17 (6.89)	0.298

Based on the mixed effect regression model, the hatchability rate was significantly lower in LD2 [β (SE) = -16.17 (2.64), P < 0.001], LD3 [β (SE) = -5.83 (2.64), P = 0.027], and LD4 [β (SE) = -5.83 (2.64), P = 0.027] compared to LD1 in the uninfected *Ae. aegypti* strains. Similarly, for the *w*AlbB (Pud) strain, the hatchability rate was significantly lower in LD2 [β (SE) = -13.33 (1.94), P < 0.001] and LD3 [β (SE) = -15.50 (1.94), P < 0.001], but not significantly different in LD4 [β (SE) = 2.50 (1.94), P = 0.197] compared to LD1. For the *w*Mel strain, hatchability was significantly lower in LD2 [β (SE) = -48.67 (1.48), P < 0.001] and LD3 [β (SE) = -34.67 (1.48), P < 0.001], and significantly higher in LD4 [β (SE) = -7.83 (1.48), P < 0.001] relative to LD1.The diet with the highest hatchability is recommended for mass rearing in *Wolbachia*-based vector control. While all diets showed significant effects, only LD3 had a negative biological impact on hatchability.

#### 3.1.2 Pupation

The pupation rate was significantly lower in LD2 and LD3 compared to LD1 in the uninfected *Ae. aegypti* strains, while LD4 showed no significant difference. For the *w*AlbB (Pud) strain, the pupation rate was significantly lower in LD2 but not significantly different in LD4 compared to LD1. For the *w*Mel strain, pupation was significantly lower only in LD2 showed no significant differences relative to the LD1diet.

In the F0 generation, pupation ranged from 95.7% to 99.0% across the four diets in uninfected *Ae. aegypti.* For the *w*AlbB (Pud) strain, the highest pupation was observed in LD1 (97.3%), followed closely by LD2 (97.0%). In the *w*Mel (Pud) strain, pupation was highest in LD2 (98.0%) and LD4 (96.7%). In the F1 generation, pupation ranged from 85.0% (LD3) to 97.3% (LD1) in uninfected *Ae. aegypti*, from 85.0% (LD3) to 99.0% (LD1) in the *w*AlbB (Pud) strain, and from 78.0% (LD2) to 99.3% (LD4) in the *w*Mel (Pud) strain.

The pupation rate was significantly lower in LD2 [β (SE) = -5.00 (1.89), P = 0.008] and LD3 [β (SE) = -7.50 (1.89), P < 0.001] compared to LD1 in the uninfected *Ae. aegypti* strains, while LD4 showed no significant difference [β (SE) = -2.33 (1.89), P = 0.218]. For the *w*AlbB (Pud) strain, the pupation rate was significantly lower in LD2 [β (SE) = -7.17 (2.11), P = 0.001] and LD3 [β (SE) = -9.67 (2.11), P < 0.001], but not significantly different in LD4 [β (SE) = -1.33 (2.11), P = 0.528] compared to LD1. For the *w*Mel strain, pupation was significantly lower only in LD2 [β (SE) = -8.50 (3.23), P = 0.009], while LD3 [β (SE) = -1.33 (3.23), P = 0.680] and LD4 [β (SE) = 1.33 (3.23), P = 0.680] showed no significant differences relative to the LD1diet.

#### 3.1.3 Adult emergence

The adult emergence rate was significantly higher in the LD1& LD4 uninfected *Ae. aegypti* strains. For the *w*AlbB (Pud) strain, it was significantly lower in LD2 and LD3 but did not differ significantly different in LD4 compared to LD1. For the *w*Mel strain, adult emergence was significantly higher in the LD1 diet. In the F0 generation, adult emergence ranged from 95.7% to 99.0% across the four diets in uninfected *Ae. aegypti*. For the *w*AlbB (Pud) strain, the highest adult emergence was observed in LD1 (97.3%) and LD2 (96.3%), followed by LD4 (96.0%). In the *w*Mel (Pud) strain, adult emergence was highest in LD2 (97.0%) and LD4 (96.7%). In the F1 generation, adult emergence ranged from 85.0% (LD3) to 96.0% (LD4) in uninfected *Ae. aegypti*, from 85.0% (LD2) to 98.0% (LD1) in the *w*AlbB (Pud) strain, and from 78.0% (LD2) to 96.3% (LD1 and LD3) in the *w*Mel (Pud) strain. Based on the mixed effect regression model, the adult emergence rate was significantly lower in LD2 [β (SE) = -4.00 (1.85), P = 0.031] and LD3 [β (SE) = -6.50 (1.85), P < 0.001] compared to LD1 in the uninfected *Ae. aegypti* strains, while LD4 showed no significant difference [β (SE) = -1.33 (1.85), P = 0.472]. For the *w*AlbB (Pud) strain, the adult emergence rate was significantly lower in LD2 [β (SE) = -7.00 (2.02), P = 0.001] and LD3 [β (SE) = -9.33 (2.02), P < 0.001], but not significantly different in LD4 [β (SE) = -0.83 (2.02), P = 0.679] compared to LD1. For the *w*Mel strain, adult emergence was significantly lower only in LD2 [β (SE) = -8.00 (3.08), P = 0.009], while LD3 [β (SE) = -0.17 (3.08), P = 0.957] and LD4 [β (SE) = 0.00 (3.08), P = 0.999] showed no significant differences relative to the LD1 diet.

#### 3.1.4 Male–female ratio

The male-to-female ratio of emerged adults was recorded for each diet across all *Ae. aegypti* strains. Males consistently outnumbered females across all diets and mosquito strains. Female percentage ranged from 47.8-49.5% (F0) and 48.3-49.0% (F1) in uninfected *Ae. aegypti*. For *w*AlbB (Pud), the highest female percentages were LD1 (49.7%) in F0 and LD2 (47.9%) in F1. For *w*Mel (Pud), the highest were LD2 (49.1%) in F0 and LD1 (48.5%) in F1. The female percentage showed no significant differences in uninfected *Ae. aegypti*: LD2 [β=-1.09(0.63), P = 0.084], LD3 [β=-0.88(0.63), P = 0.160], LD4 [β=-0.70(0.63), P = 0.262]. Similarly, *w*Mel strain showed no effects: LD2 [β=-0.46(0.50), P = 0.356], LD3 [β=-0.28(0.50), P = 0.573], LD4 [β=-0.50(0.50), P = 0.310]. However, the *w*AlbB strain had a significant reduction in LD3 [β=-2.09(1.04), P = 0.045].

#### 3.1.5 Fecundity

**Figure 2:**
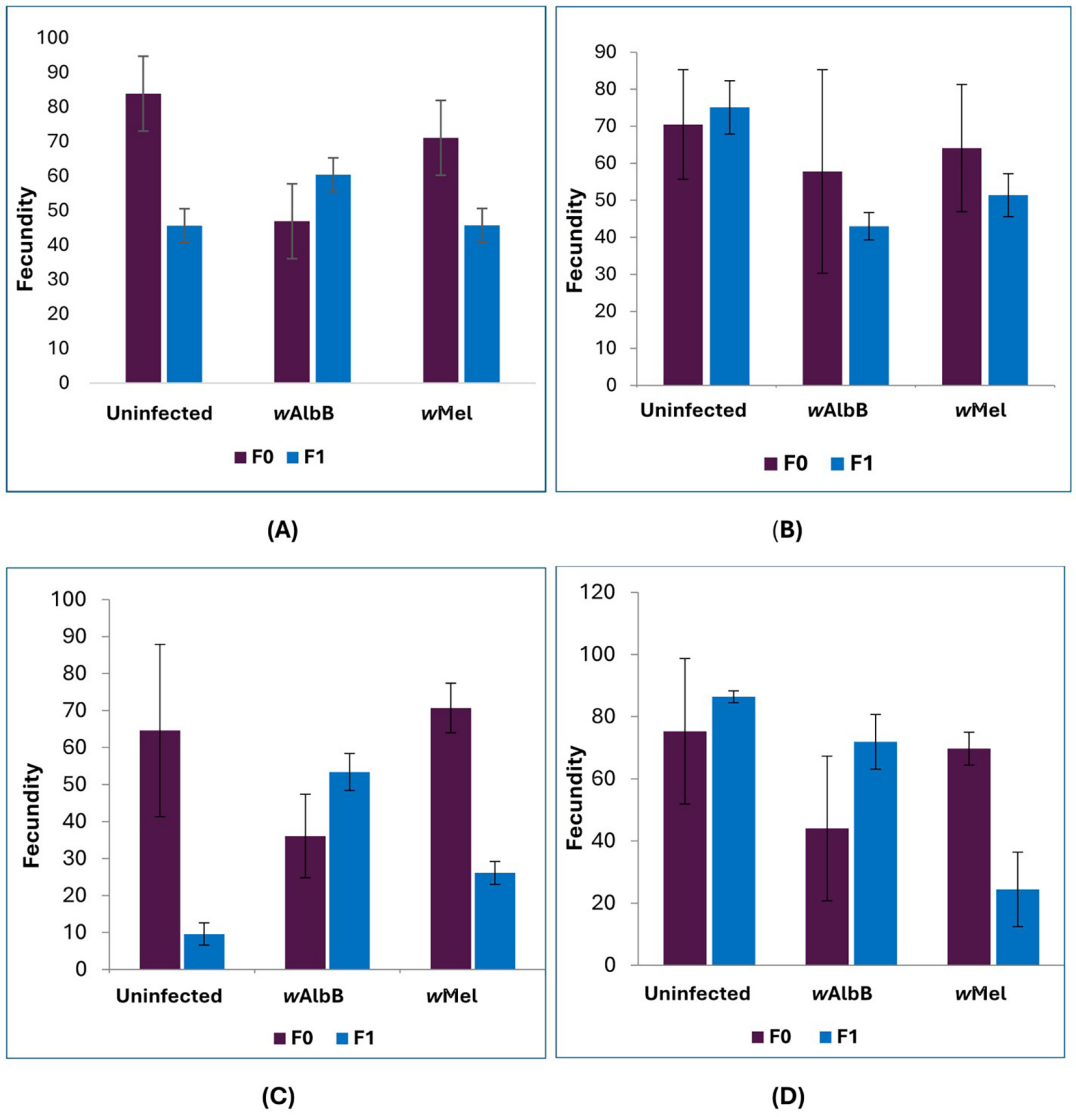
Fecundity (mean ± SE) of uninfected, *w*AlbB, and *w*Mel strains of *Ae. aegypti* (Puducherry strain) females across two generations (F0 and F1) under four larval diets: **(A)** LD1 – Fish feed; **(B)** LD2 – Laboratory rodent diet; **(C)** LD3 – Mushroom powder; **(D)** LD4 – Dog biscuit with Brewer’s yeast (3:2). Each bar represents the mean fecundity from three replicates of 100 larvae per mosquito line per diet (n = 3 replicates per group). Error bars indicate the standard error of the mean, representing the variability of the sample estimate for each group.

Fecundity was significantly lower only in LD3 compared to LD1 in the uninfected *Ae. aegypti* strains, while LD2 and LD4 showed no significant differences. For both *Wolbachia*-infected strains, no significant dietary effects on fecundity were observed. The fecundity ranged from 68.8 to 83.9 eggs per female across the four diets in uninfected *Ae. aegypti* in the F0 generation (**Figure 2**). For the *w*AlbB (Pud) strain, the highest fecundity was observed in LD2 (61.8 eggs), followed by LD1 (46.9 eggs) and LD4 (42.0 eggs). In the *w*Mel (Pud) strain, fecundity was highest in LD4 (78.0 eggs) and LD3 (72.7 eggs). In the F1 generation, fecundity ranged from 9.6 (LD3) to 86.4 (LD4) eggs per female in uninfected *Ae. aegypti*, from 43.0 (LD2) to 71.9 (LD4) in the *w*AlbB (Pud) strain, and from 24.5 (LD4) to 51.4 (LD2) in the *w*Mel (Pud) strain. The fecundity was significantly lower only in LD3 [β (SE) = -25.50 (11.11), P = 0.022] compared to LD1 in the uninfected *Ae. aegypti* strains, while LD2 [β (SE) = 8.46 (11.11), P = 0.446] and LD4 [β (SE) = 15.81 (11.11), P = 0.155] showed no significant differences. For both *Wolbachia*-infected strains, no significant dietary effects on fecundity were observed. In the *w*AlbB (Pud) strain: LD2 [β (SE) = -1.15 (9.40), P = 0.903], LD3 [β (SE) = -9.23 (9.40), P = 0.326], and LD4 [β (SE) = 3.44 (9.40), P = 0.715]. Similarly, for the *w*Mel strain: LD2 [β (SE) = 0.21 (6.89), P = 0.976], LD3 [β (SE) = -9.06 (6.89), P = 0.189], and LD4 [β (SE) = -7.17 (6.89), P = 0.298] all remained non-significant compared to the LD1 diet.

#### 3.1.6 Adult survival

**Figure 3:**
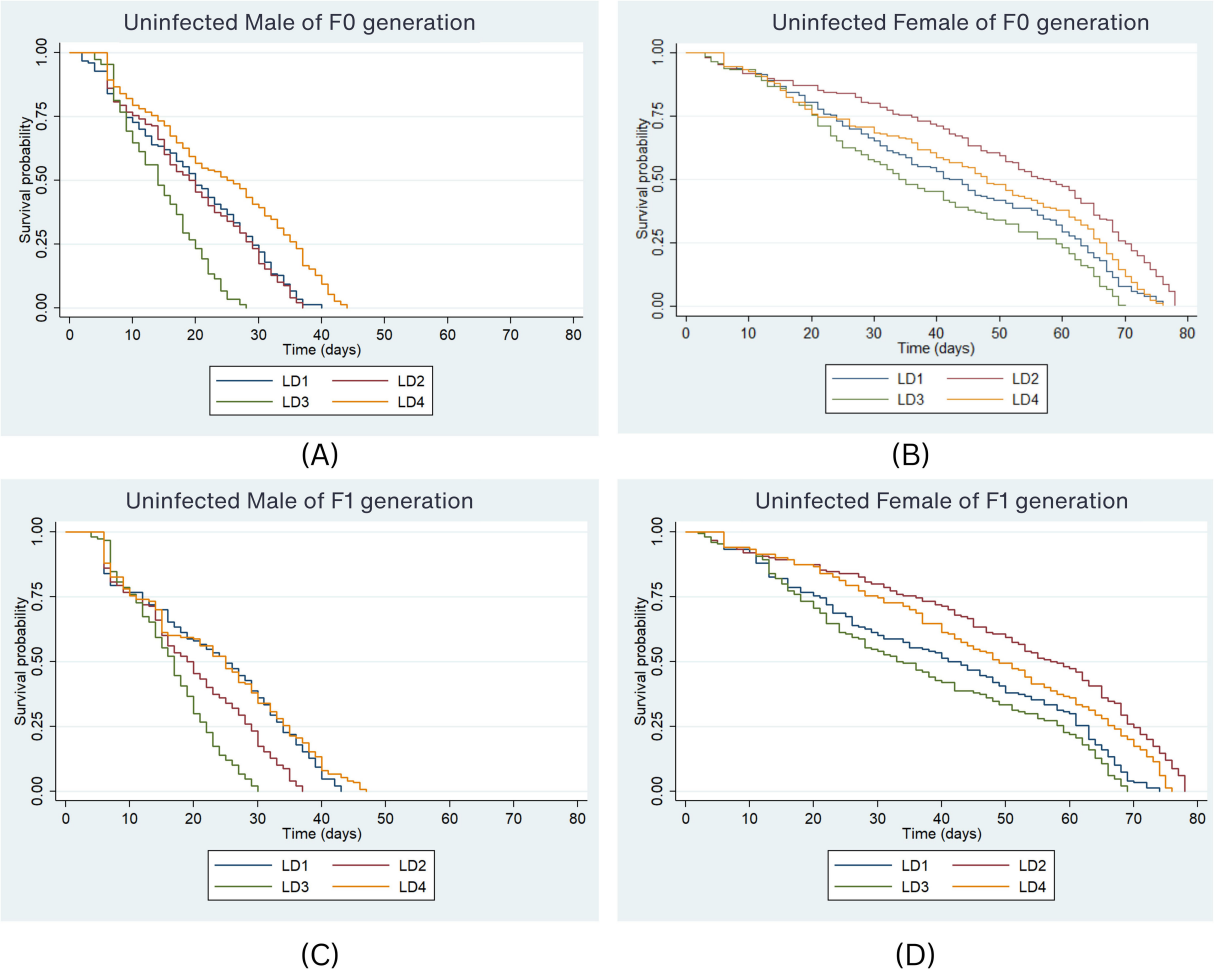
Survival probability of uninfected *Ae. aegypti* male and female in F0 **(A, B)** & F1 **(C, D)** generation under the four larval diets. Kaplan-Meier survival curves were graphically presented to compare the impact of different diets on survival. The log-rank test was used to compare the median survival times (50%) among lines and the Cox proportional hazards model was employed, providing hazard ratios (HR) and 95% confidence intervals separately for males and females.

The impact of different diets on adult mosquito survival over time was examined. In the F0 generation, the median survival duration of uninfected male *Ae. aegypti* was 20 days under the LD1 diet (**Figure 3A**), i.e., 50% of males survived for 20 days on LD1. Under the LD2 diet, the median survival was 19 days, while in LD3 and LD4, 50% of males survived for 14 and 25 days, respectively. Survival curves significantly differed between larval diets for uninfected males (P < 0.001, Log-rank test). The risk of death was significantly higher in LD3 (HR = 2.03, P < 0.001) compared to LD1, while significantly lower mortality was observed in LD4 (HR = 0.55, P < 0.001) compared to LD1. Among females, the lowest median survival was 34 days under LD3, whereas median survival was 42, 57, and 48 days under LD1, LD2, and LD4, respectively (**Figure 3B**). The risk of death was significantly lower in LD2 (HR = 0.56, P < 0.001) compared to all other diets. No significant difference was observed for LD3 (HR = 1.35, P = 0.074) or LD4 compared to LD1.

In the F1 generation, 50% of males survived up to 25 days under LD1 and LD4 (**Figure 3C**). Survival probabilities differed significantly among larval diets (P < 0.001, Log-rank test). The risk of death was significantly higher in LD2 (HR = 1.61, P < 0.001) and LD3 (HR = 2.47, P < 0.001) compared to LD1. No significant difference was observed for LD4 (HR = 0.89, P = 0.333) compared to LD1. Among females, median survival was 41 days under LD1, while 50% survived up to 57, 33, and 49 days in LD2, LD3, and LD4, respectively (**Figure 3D**). The risk of death was highest under LD3 in both males and females across generations (HR = 1.58, 95% CI: 1.41–1.78, P < 0.001). Compared to females, males consistently had a higher risk of death across both generations (HR = 5.35, 95% CI: 4.79–5.97, P < 0.001). However, no significant difference in mortality risk was observed between generations in the uninfected *Ae. aegypti* strain.

**Figure 4:**
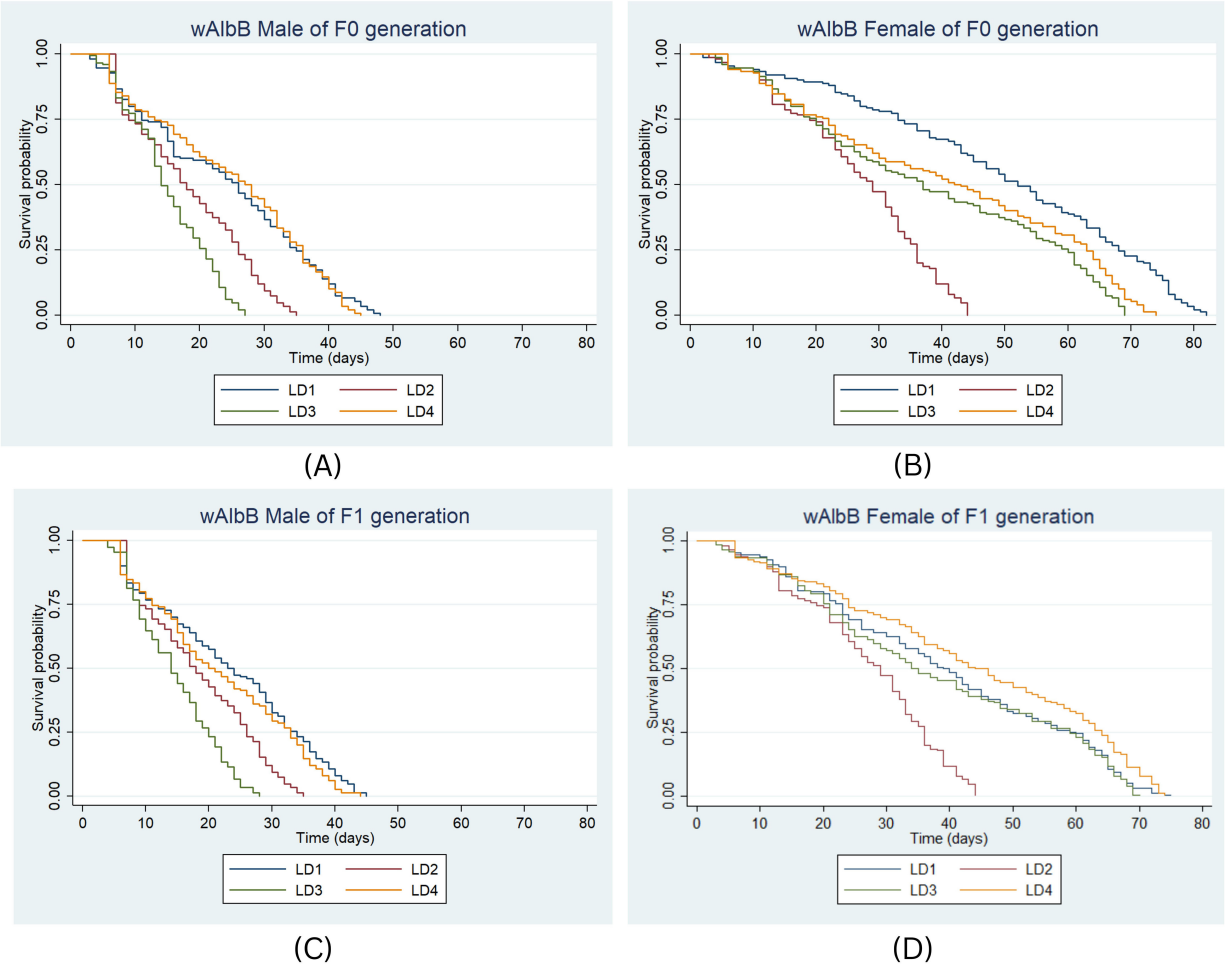
Survival probability of *w*AlbB male and female in F0 **(A, B)** & F1 **(C, D)** generation under the four larval diets. Kaplan-Meier survival curves were graphically presented to compare the impact of different diets on survival. The log-rank test was used to compare the median survival times (50%) among lines and the Cox proportional hazards model was employed, providing hazard ratios (HR) and 95% confidence intervals separately for males and females.

For the *w*AlbB strain in the F0 generation, 50% of males survived for 27 days in LD4 and 26 days in LD1 (**Figure 4A**). The risk of death was significantly higher in LD2 (HR = 2.25, P < 0.001) and LD3 (HR = 3.51, P < 0.001) compared to LD1, with no significant difference in LD4 (HR = 1.03, P = 0.80). Among females, the highest median survival was 52 days under LD1, followed by 42 and 37 days in LD4 and LD3, respectively (**Figure 4B**). Significant differences were observed under LD2 (HR = 4.88, P < 0.001), LD3 (HR = 2.15, P < 0.001), and LD4 (HR = 1.76, P < 0.001) compared to LD1.

In F1, 50% of *w*AlbB males survived for 23 days under LD1 (**Figure 4C**). Median survival was 18 days in LD2, and 14 and 20 days in LD3 and LD4, respectively. Survival curves differed significantly across larval diets (P < 0.001, log-rank test). The risk of death was significantly higher in LD2 (HR = 1.97, P < 0.001) and LD3 (HR = 3.15, P < 0.001), but not in LD4 (HR = 1.2, P = 0.11) compared to LD1. Among females, median survival was highest in LD4 (44 days) (**Figure 4D**). The risk of death was significantly higher in LD2 (HR = 2.50, P < 0.001), while significant differences were also recorded in LD2 (HR = 2.50, P < 0.001) and LD4 (HR = 0.77, P < 0.001) compared to LD1. Overall, the risk of death was significantly higher among males under LD2 (HR = 2.36, 95% CI: 2.09–2.66, P < 0.001) and LD3 (HR = 1.94, 95% CI: 1.72–2.18, P < 0.001). Lower mortality risk (HR < 1) was observed under LD1 and LD4, but not statistically significant (P > 0.001). Males exhibited significantly higher mortality than females across both generations (HR = 3.45–4.21, P < 0.001), though no significant variation was observed between generations (HR = 1.13, 95% CI: 1.04–1.22, P > 0.001).

**Figure 5:**
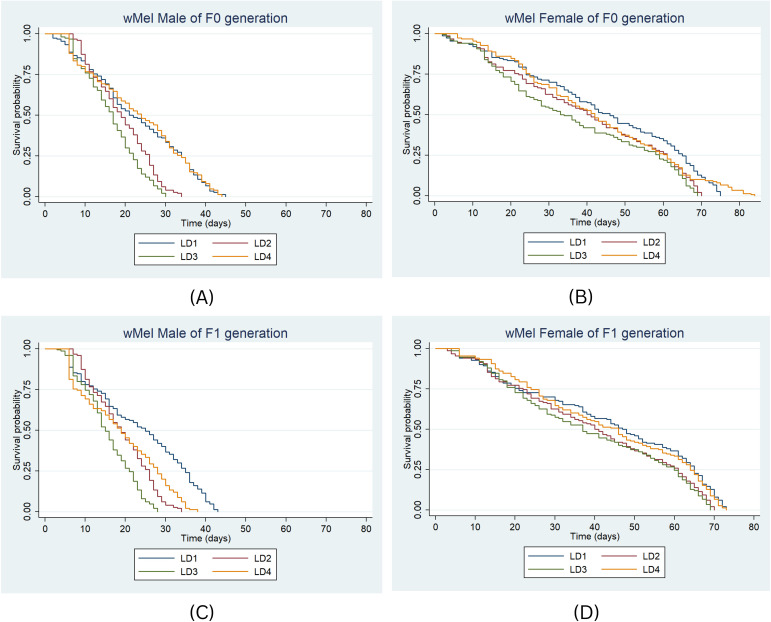
Survival probability of *w*Mel male and female in F0 **(A, B)** & F1 **(C, D)** generation under the four larval diets. Kaplan-Meier survival curves were graphically presented to compare the impact of different diets on survival. The log-rank test was used to compare the median survival times (50%) among lines and the Cox proportional hazards model was employed, providing hazard ratios (HR) and 95% confidence intervals separately for males and females.

For the *w*Mel strain in F0, median survival durations for males differed significantly across diets: LD2 (HR = 1.94, P < 0.001), LD3 (HR = 2.53, P < 0.001), and LD4 (HR = 0.97, P < 0.001) compared to LD1 (**Figure 5A**). For females, the highest median survival was 46 days under LD1 and the lowest was 33 days under LD3 (**Figure 5B**). Significant differences were found for LD2 (HR = 1.51, P < 0.001) and LD3 (HR = 1.53, P < 0.001) compared to LD1.

In the F1 generation, 50% of *w*Mel males survived for 25 days under LD1 (**Figure 5C**). Median survival was 19 days in LD2, and 15 and 19 days in LD3 and LD4, respectively. The risk of death was significantly higher in LD2 (HR = 2.3, P < 0.001), LD3 (HR = 3.5, P < 0.001), and LD4 (HR = 1.9, P = 0.11) compared to LD1. For females, median survival durations were 47 days in LD1, and 40, 37, and 46 days in LD2, LD3, and LD4, respectively (**Figure 5D**). The risk of death was significantly higher under LD3 (HR = 1.5, P < 0.001) and LD2 (HR = 1.4, P < 0.001), while a significant difference was not observed for LD4 (HR = 1.08, P = 0.47) compared to LD1.

In summary, among the *w*Mel strains, the death risk was significantly higher under LD2 (HR = 1.49, 95% CI: 1.33–1.67, P < 0.001) and LD3 (HR = 1.83, 95% CI: 1.63–2.05, P < 0.001). Males consistently exhibited higher mortality than females (HR = 4.83, 95% CI: 4.34–5.38, P < 0.001), whereas no significant difference was observed between generations (HR = 1.06, 95% CI: 0.98–1.15, P > 0.001).

## 4 Discussion

Sustainable vector control tools are consistently in demand to suppress vector populations and prevent vector-borne diseases. Integrated Vector Control Management (IVM) is a comprehensive approach that ensures the sustainability of vector control methods, explicitly targeting the control of dengue vectors (38). Trans infection of symbiotic bacteria into a targeted vector and releasing them into the field for population replacement/suppression is one of these comprehensive approaches to tackling vector populations (39, 40). It is a long-term solution and a promising tool for controlling *Aedes* mosquitoes, mainly *Ae. aegypti*, the principal vector of major arboviral diseases. Continuous use of insecticides has resulted in resistance among the mosquito population and has made them no longer effective against the vectors. Hence, the *Wolbachia-*based vector control strategy offers an alternative that may be less prone to resistance issues, helping maintain effective control measures in the long run (41).

Multiple countries have successfully released *Wolbachia*-transinfected *Aedes* mosquitoes for population replacement, with the World Mosquito Program introducing *Wolbachia* in 14 countries (42). The *w*Mel and *w*AlbB strains have been successfully established in wild *Ae. aegypti* populations in Australia and Malaysia (43–45). Laboratory studies demonstrate a notable reduction in virus transmission capacity, particularly for dengue (8, 46, 47). *Wolbachia* infection enhances diet-related nutritional stress, reducing dengue susceptibility (48) and competes with pathogens for nutrients, inhibiting replication and shortening vector lifespan (49, 50). Field studies following releases confirm effectiveness, with mass releases of *Wolbachia*-transinfected males significantly reducing egg hatch rates through cytoplasmic incompatibility and decreasing Zika virus transmission compared to control areas (16, 51).

Field release of *Wolbachia*-transinfected *Ae. aegypti* requires fitness evaluation, as successful spread depends on efficient maternal transmission without substantial fitness costs (46, 47). Sadanandane et al. (31) assessed *w*Mel (Pud) and *w*AlbB (Pud) strain fitness, examining life-history parameters, infection persistence, maternal transmission, cytoplasmic incompatibility, and insecticide susceptibility. Proper larval nutrition is crucial for the mass release of competent mosquitoes and high-quality transinfected females (52, 53).

The two primary dietary formulations are widely utilized in mosquito laboratory studies: The IAEA-standardized diet and formulations based on commercial fish food products like TetraMin. Despite of this, previous studies evaluated different larval diets for their effect on *Ae. aegypti* fitness. In Mexico, protein-based diets such as tilapia fish food, bovine liver powder, and porcine meal positively affected *Wolbachia*-infected *Ae. aegypti* development (28). Similarly, a study conducted in Sri Lanka revealed that a combination of dry fish powder and brewer’s yeast enhanced larval development, increased reproductive output, and extended male lifespan when compared to the standard IAEA dietary formulation (29). Correspondingly, Bond et al. (21) found that laboratory rodent feed formulations resulted in larger adult mosquitoes and enhanced fecundity, attributed to their elevated carbohydrate composition. Conversely, replacing animal-derived proteins with plant-based protein sources in larval nutrition negatively affected egg viability, extended developmental periods, and diminished reproductive performance (30). A recent study by Yatim et al. (54) revealed that uninfected mosquito strains demonstrated superior overall fitness performance across all tested dietary formulations, including plant-based diet, Khan diet, fish food, and IAEA diet, compared to *w*MelM and *w*AlbB trans-infected strains. While previous studies have compared established larval diet formulations such as IAEA, Khan diet, and fish feed-based diets for mass production of *Ae. aegypti*, the current study optimized practical larval dietary approaches for scaling up mass production of *Wolbachia* transinfected *Ae.aegypti* (*w*Mel and *w*AlbB) strains in resource-limited settings. The current study evaluated four commercially available larval diets: fish feed (LD1), laboratory rodent diet (LD2), mushroom powder (LD3), and dog biscuits with brewer’s yeast (LD4) on two *Wolbachia*-transinfected strains compared to uninfected *Ae. aegypti*. Fish feed (TetraBits) and dog biscuits with brewer’s yeast are routinely used at ICMR-VCRC, while mushroom powder was included despite a higher cost to compare plant versus animal protein effects. The study examined two generations (F0 and F1) to understand how larval nutrition influences adults and progeny, evaluating life table characteristics including hatchability, pupation rate, adult emergence, sex ratio, fecundity, and survival.

### 4.1 Effect of larval diets on life table traits

Fish feed (LD1) and dog biscuit with yeast (LD4) significantly impacted hatchability of the three *Ae. aegypti* strains compared to other diets, with no significant difference between generations (F1 & F2). High egg hatchability is crucial for mass rearing, ensuring sufficient viable larvae for mosquito production (55). These findings line up with Salim et al. (30), who reported the highest hatchability with the IAEA diet containing fish meal (73.9%) and the lowest with mushroom powder (40.7%), while Bond et al. (21) found higher hatchability (~80%) with the laboratory rodent diet compared to the IAEA diet.

A larger number of pupae would contribute to a higher number of adult emergences, a key parameter in the life table of the mosquito (30). Similar to hatchability, the diets, LD1 and LD4, had a significant impact, resulting in a higher percentage of pupation in all *Ae. aegypti* strains. We recorded the highest pupation rate for the *w*AlbB strain under LD1 and LD4. The *w*Mel strain also showed higher pupation under LD1 and LD4. However, the uninfected *Ae. aegypti* strain showed a comparatively lower percentage of pupation in all the diets. In a recent study by Yatim et al. (2025), it was found that uninfected mosquito strains demonstrated superior overall fitness performance across all tested dietary formulations, including a plant-based diet, Khan diet, fish food, and IAEA diet, compared to *w*MelM and *w*AlbB infected strains. On the contrary, Contreras-Perera et al. (28) observed no significant differences between the percentage pupation of *Wolbachia*-transinfected and wild-type lab-established strain, *Ae. aegypti* at the different diets used in their study.

In an impact study comparing Khan’s and IAEA-2 larval diets, the IAEA-2 diet resulted in higher pupation rates and produced larger adult mosquitoes in *Wolbachia*-uninfected *Ae. aegypti*, with improved male longevity (56). Their findings demonstrate that both diets have significant positive effects across all the life table traits, making them suitable for sterile insect technique applications. Similarly, Gunathilaka et al. (57) reported the highest pupation rate at the highest larval feed concentration compared to the lowest, supporting the significant influence of larval nutritional resources on pupation success.

Adult emergence in the three mosquito strains was proportional to pupation rates. Under the LD1 diet, *Wolbachia*-transinfected strains (*w*Mel and *w*AlbB) showed higher emergence than uninfected *Ae. aegypti* in F1 generation, with the opposite trend in F0 generation, though differences were not statistically significant. LD1 and LD4 diets produced no significant variation between strains or generations, achieving >90% emergence across all three strains. On the other hand, LD2 and LD3 diets showed significant differences between strains and generations. Males consistently outnumbered females across all strains and diets, but larval diets did not significantly affect the male-to-female ratio in either generation. This consistent sex ratio across diets supports *Wolbachia* population replacement strategies, which require equal proportions of both sexes for mass rearing. These findings contrast with Bond et al. (21), who reported higher male proportions under the IAEA diet compared to the laboratory rodent diets.

Significant variability in hatchability, pupation, adult emergence, male–female ratio, fecundity, and adult survival among *Wolbachia*-transinfected *Ae. aegypti* strains and diets is primarily explained by differences in larval diet composition and mosquito infection status. Larval diets that are balanced in nutrients (such as LD1 and LD4) support higher hatchability and overall fitness, likely due to optimal protein and carbohydrate ratios, which enhance development and egg viability. In contrast, diets deficient or imbalanced in nutrients (such as LD3 and LD2) can restrict larval development, reduce energy reserves, and lead to lower hatchability, pupation, and survival rates.​ Wolbachia infection status also contributes to observed variability, as different strains respond distinctly to dietary composition, influencing life table parameters beyond diet alone. The interplay of genetic background, nutrient intake, and microbial symbionts (Wolbachia) underscores the biological basis for significant differences in mosquito population outcomes across experimental groups. Thus, both dietary quality and infection status are key drivers of the significant variability observed in *Ae. aegypti* life table parameters.

### 4.2 Influence on mosquito reproductive traits and survival

A significant positive effect on fecundity was observed among mosquito strains under LD1 and LD4 diets, supporting the importance of diet composition in influencing reproductive fitness. In contrast, LD2 and LD3 diets failed to produce a significant impact, likely due to suboptimal nutritional content or poorer assimilation efficiency. These findings are consistent with earlier observations by Sadanandane et al. (31), who reported higher fecundity in *w*AlbB compared to both wild-type and *w*Mel strains when reared under fish feed diets. A similar study also observed that *w*Mel strains persistently had low fecundity compared to *w*AlbB and the uninfected strains of *Ae. aegypti* in all the diets (Plant-based, Khan’s, Fish feed and IAEA diets) tested (Yatim et al, 2025). Our study results revealed that the fecundity of all the mosquito strains was high under LD1 and LD2 diets, and the *w*AlbB strain of *Ae. aegypti* showed comparatively high fecundity under all the diets. Collectively, these results underscore the interplay between *Wolbachia* strain, generation, and larval diet in shaping reproductive capacity. Importantly, the improved fecundity of transinfected strains such as *w*AlbB under specific diet conditions suggests a potential pathway for optimizing mass-rearing protocols for the release of *Wolbachia*-transinfected *Ae. aegypti* into the field.

The highest survival was observed in uninfected females reared on the LD2 diet, with a lifespan of 57 days in both the F0 and F1 generations, indicating that this diet provides optimal nutritional support for adult longevity. In contrast, the LD3 diet resulted in the shortest survival (33 days) for both uninfected and *w*Mel-infected females, reflecting its inadequate nutritional composition. Notably, the LD1 and LD4 diets significantly increased median survival in uninfected F0 males, highlighting the protective effects of these protein-rich diets. Similarly, Sasmita et al. (56) demonstrated that both Khan’s and IAEA-2 larval diets significantly enhanced adult body size and survival parameters, supporting their suitability for SIT applications. Furthermore, Gunathilaka et al. (57) found that increasing larval food supply resulted in adults with higher fecundity and greater survival rates, underscoring the critical influence of nutritional quality and quantity on mosquito life-history traits.

### 4.3 Implications for *Wolbachia* transmission and vector control

The study highlights cost-effective larval diets for mass rearing *Wolbachia*-transinfected and uninfected *Ae. aegypti*. Fish feed (LD1) and dog biscuit with brewer’s yeast (LD4) significantly improved life table traits compared to mushroom powder and laboratory rodent diets, highlighting the importance of protein-rich nutrition for larval development and survival (29, 58). These findings support adopting a low-cost diet for Indian mass-rearing facilities. A balanced mosquito diet enhances *Wolbachia* density and stability within the mosquitoes, strengthening pathogen blocking. Diet quality throughout a mosquito’s life influences adult size, lifespan, and biting behavior, and pathogen transmission capacity (59). The diet with significant effects is recommended for mass rearing in *Wolbachia*-based vector control. However, statistical significance alone doesn’t guarantee practical benefits. While all diets showed significant effects, only LD3 had a negative biological impact on hatchability. This highlights the importance of considering both statistical and biological relevance in diet selection for vector control programs. This study validated suitable larval diets for mass rearing transinfected mosquitoes, with life table traits significantly impacted by recommended diets. Mosquito survival and wild population establishment for *Wolbachia* dissemination is fundamental for successful vector control strategies, potentially contributing to reduced dengue and arboviral disease burden as a promising control tool.

### 4.4 Limitations of the study

The limitation of this study is the absence of formal sample size calculation and power analysis. As an exploratory experimental study designed to screen and compare artificial larval diets, the primary goal was to assess feasibility and biological plausibility rather than detect a pre-specified effect size. Following WHO guidelines for bioassay, 100 third-instar larvae per replicate were used for each of the three *Wolbachia* strains. This approach may limit the statistical power and generalizability of the findings, which will be addressed in future, more comprehensive studies.

## 5 Conclusion

This study demonstrates that fish feed (LD1) and dog biscuit with brewer’s yeast (LD4) are superior, cost-effective larval diets for mass-rearing *Wolbachia*-transinfected *Ae. aegypti*. These protein-rich diets significantly enhanced critical life table parameters; hatchability, pupation rates, adult emergence, fecundity, and survival across both infected and uninfected strains over two generations. The *w*AlbB strain showed particularly robust performance under these optimal dietary conditions. The results of this study suggest that LD1 (fish feed) can be recommended as the superior larval diet for the mass rearing of *Wolbachia*-transinfected strains, although both LD1 and LD4 diets demonstrated positive effects on all the *Ae. aegypti* strains. Meanwhile, LD4 (dog biscuit + brewer’s yeast) can be recommended for the routine rearing of uninfected *Ae. aegypti* colonies, as it is comparatively cost-effective and readily available in India. These findings could contribute to the large-scale mosquito rearing programs under the *Wolbachia* strategy, ultimately supporting the implementation of sustainable vector control approaches for arboviral disease management.

## Data Availability

The original contributions presented in the study are included in the article/**Supplementary Material**. Further inquiries can be directed to the corresponding author.

## Supplementary material

Supplementary Table 1 Larval diets and their composition (As per manufacturer’s standard).

Supplementary File 1 Mixed-effect linear regression model results.

Supplementary File 2 Raw data of mosquito survival in each stage under different diets (F0 & F1 generation).
