# Supplementary material for: Life table variations in Wolbachia-transinfected (wMel & wAlbB strains) and uninfected Aedes aegypti: the role of various larval diets
Source: Front Insect Sci. 2025 Dec 12;5:1679816. doi: 10.3389/finsc.2025.1679816 (PMC12741118; doi:10.3389/finsc.2025.1679816)
Supplement: Supplementary Table 1 — Larval diets and their composition (As per manufacturer’s standard). [file Table1.docx]

**Supplementary Table 1. Larval diets and their composition (As per manufacturer’s standard)**

| **S. No** | **Diets** | **Composition** | **Nutritional value** |
| --- | --- | --- | --- |
| 1 | LD 1 | Mollusks and crustaceans, Fish and fish derivatives, Yeasts, Algae, Vegetable protein extracts, Oils and fats, Various sugars (Oligofructose 1%), Cereals, Minerals | Crude protein, crude fat, Carbohydrate, crude fibre, moisture content, vitamin D3, vitamin A, trace elements (Mn, Zn & Fe), and antioxidants. |
| 2 | LD 2 | Maize, Beet pulp, Alfalfa meal, Salt, Fish meal, Oats, Brewer's yeast, Cane molasses, Whey, Porcine meat meal, Soybean meal, Wheat, and a mixture of Vitamins and Minerals | Carbohydrate, Crude protein, crude fat, crude fibre, moisture content, Nitrogen free extract, vitamins, minerals and sodium. |
| 3 | LD 3 | 100% Natural button mushroom | Carbohydrate, crude protein, dietary fibre, fat, iron, calcium, vitamin A& C and sodium (Krishnamoorthi et al., 2022) |
| 4 | LD 4 | Dog biscuit and Brewer's yeast in the rate of (3:2)   - Dog biscuit- meat and meat by-products, chicken and chicken by-products, vegetables and vegetable by-products, - Salt, vegetable oils, milk powder, iodized salt, cereals and cereal by-products, and antioxidants, essential vitamins, and minerals. | Brewer's yeast- crude protein, crude fat, crude ash, , crude fibre, sugar and starch, vitamins and minerals  Dog biscuit-Crude fat, crude fiber and crude protein, calcium, vitamins & and minerals |
