# Supplementary material for: Life table variations in Wolbachia-transinfected (wMel & wAlbB strains) and uninfected Aedes aegypti: the role of various larval diets
Source: Front Insect Sci. 2025 Dec 12;5:1679816. doi: 10.3389/finsc.2025.1679816 (PMC12741118; doi:10.3389/finsc.2025.1679816)
Supplement: Supplementary File 1 — Mixed-effect linear regression model results. [file Table2.docx]

**Table S2: A mixed-effects linear regression model for hatchability of uninfected *Ae. aegypti* under the four diets.**

| **Effect type** | **Source** | **Estimate** | **95% CI** | **p-value** |
| --- | --- | --- | --- | --- |
| Fixed | Intercept | 94.67 | 90.78, 98.55 | <0.001 |
|  | LD2 | -16.17 | -21.34, -10.99 | <0.001 |
|  | LD3 | -5.83 | -11.00, -0.66 | 0.027 |
|  | LD4 | -5.83 | -11.00, -0.66 | 0.027 |
| Random | Generation (Var) | 0.90 | 0.00, 313.94 | - |
|  | Replicate within generation (Var) | 1.01e-09 | 1.39e-23, 74038.43 | - |
| Residual variance |  | 20.87 | 11.56, 37.68 | - |
| Model fit | AIC =155.86, BIC =164.11 | - | - | - |
| ICC | Generation=0.04, Replicate=0.04 | - | - | - |
| R2 (marginal) | 0.62 | - | - | - |
| R2 (Conditional) | 0.64 | - | - | - |
| Wald chi-square, P-value | 39.03, P<0.001 | - | - | - |
| LR test, P-value | 0.18, P = 0.914 | - | - |  |

**Pairwise comparisons of marginal linear predictions**

Margins : asbalanced

----------------------------------------------------------------------------------

| Unadjusted Unadjusted

| Contrast Std. Err. z P>|z| [95% Conf. Interval]

-----------------+----------------------------------------------------------------

nooflarvaemerged |

diet1 |

LD2 vs LD1 | -16.16667 2.637505 -6.13 0.000 -21.33608 -10.99725

LD3 vs LD1 | -5.833333 2.637505 -2.21 0.027 -11.00275 -.6639182

LD4 vs LD1 | -5.833333 2.637505 -2.21 0.027 -11.00275 -.6639182

LD3 vs LD2 | 10.33333 2.637505 3.92 0.000 5.163918 15.50275

LD4 vs LD2 | 10.33333 2.637505 3.92 0.000 5.163918 15.50275

LD4 vs LD3 | -4.26e-14 2.637505 -0.00 1.000 -5.169415 5.169415

----------------------------------------------------------------------------------

**Table S3: A mixed-effects linear regression model for hatchability of *w*AlbB strain under the four diets.**

| **Effect type** | **Source** | **Estimate** | **95% CI** | **p-value** |
| --- | --- | --- | --- | --- |
| Fixed | Intercept | 88.00 | 84.26, 91.74 | <0.001 |
|  | LD2 | -13.33 | -17.13, -9.53 | <0.001 |
|  | LD3 | -15.50 | -19.30, -11.70 | <0.001 |
|  | LD4 | 2.50 | -1.30, 6.30 | 0.197 |
| Random | Generation (Var) | 6.65e-22 | 3.52e-46, 1255.112 | - |
|  | Replicate within generation (Var) | 10.57 | 2.49, 44.79 | - |
| Residual variance |  | 11.28 | 5.87, 21.67 | - |
| Model fit | AIC =149.60, BIC =157.85 | - | - | - |
| ICC | Generation=3.04e-23, Replicate=0.48 | - | - | - |
| R2 (marginal) | 0.75 | - | - | - |
| R2 (Conditional) | 0.87 | - | - | - |
| Wald chi-square, P-value | 133.49, P<0.001 |  |  |  |
| LR test, P-value | 6.52, P = 0.038 |  |  |  |

Pairwise comparisons of marginal linear predictions

Margins : asbalanced

----------------------------------------------------------------------------------

| Unadjusted Unadjusted

| Contrast Std. Err. z P>|z| [95% Conf. Interval]

-----------------+----------------------------------------------------------------

nooflarvaemerged |

diet1 |

LD2 vs LD1 | -13.33333 1.938881 -6.88 0.000 -17.13347 -9.533197

LD3 vs LD1 | -15.5 1.938881 -7.99 0.000 -19.30014 -11.69986

LD4 vs LD1 | 2.5 1.938881 1.29 0.197 -1.300137 6.300137

LD3 vs LD2 | -2.166667 1.938881 -1.12 0.264 -5.966803 1.63347

LD4 vs LD2 | 15.83333 1.938881 8.17 0.000 12.0332 19.63347

LD4 vs LD3 | 18 1.938881 9.28 0.000 14.19986 21.80014

----------------------------------------------------------------------------------

**Table S4: A mixed-effects linear regression model for hatchability of *w*Mel strain under the four diets.**

| **Effect type** | **Source** | **Estimate** | **95% CI** | **p-value** |
| --- | --- | --- | --- | --- |
| Fixed | Intercept | 78.33 | 75.97, 80.70 | <0.001 |
|  | LD2 | -48.67 | -51.57, -45.77 | <0.001 |
|  | LD3 | -34.67 | -37.57, -31.77 | <0.001 |
|  | LD4 | 7.83 | 4.93, 10.73 | <0.001 |
| Random | Generation (Var) | 0.718 | 0.02, 23.37 | - |
|  | Replicate within generation (Var) | 2.97e-13 | 1.80e-28, 488.87 | - |
| Residual variance |  | 6.57 | 3.63, 11.87 | - |
| Model fit | AIC =128.95, BIC =137.20 | - | - | - |
| ICC | Generation=0.10, Replicate=0.10 | - | - | - |
| R2 (marginal) | 0.99 | - | - | - |
| R2 (Conditional) | 0.99 | - | - | - |
| Wald chi-square, P-value | 2016.23, P<0.001 |  |  |  |
| LR test, P-value | 0.81, P = 0.665 |  |  |  |

Pairwise comparisons of marginal linear predictions

Margins : asbalanced

----------------------------------------------------------------------------------

| Unadjusted Unadjusted

| Contrast Std. Err. z P>|z| [95% Conf. Interval]

-----------------+----------------------------------------------------------------

nooflarvaemerged |

diet1 |

LD2 vs LD1 | -48.66667 1.479447 -32.90 0.000 -51.56633 -45.767

LD3 vs LD1 | -34.66667 1.479447 -23.43 0.000 -37.56633 -31.767

LD4 vs LD1 | 7.833333 1.479447 5.29 0.000 4.933671 10.733

LD3 vs LD2 | 14 1.479447 9.46 0.000 11.10034 16.89966

LD4 vs LD2 | 56.5 1.479447 38.19 0.000 53.60034 59.39966

LD4 vs LD3 | 42.5 1.479447 28.73 0.000 39.60034 45.39966

----------------------------------------------------------------------------------

**Table S5: A mixed-effects linear regression model for pupation of uninfected *Ae. aegypti* under the four diets.**

| **Effect type** | **Source** | **Estimate** | **95% CI** | **p-value** |
| --- | --- | --- | --- | --- |
| Fixed | Intercept | 98.17 | 94.17, 102.17 | <0.001 |
|  | LD2 | -5.00 | -8.71, -1.29 | 0.008 |
|  | LD3 | -7.50 | -11.21, -3.79 | <0.001 |
|  | LD4 | -2.33 | -6.04, 1.38 | 0.218 |
| Random | Generation (Var) | 4.74 | 0.46, 48.90 | - |
|  | Replicate within generation (Var) | 1.56e-19 | 2.16e-37, 0.11 | - |
| Residual variance |  | 10.75 | 5.94, 19.44 | - |
| Model fit | AIC =142.78, BIC =151.03 | - | - | - |
| ICC | Generation=0.31, Replicate=0.31 | - | - | - |
| R2 (marginal) | 0.35 | - | - | - |
| R2 (Conditional) | 0.55 | - | - | - |
| Wald chi-square, P-value | 17.69, P=0.0005 |  |  |  |
| LR test, P-value | 5.10, P = 0.078 |  |  |  |

Pairwise comparisons of marginal linear predictions

Margins : asbalanced

---------------------------------------------------------------------------------

| Unadjusted Unadjusted

| Contrast Std. Err. z P>|z| [95% Conf. Interval]

----------------+----------------------------------------------------------------

noofpupalformed |

diet1 |

LD2 vs LD1 | -5 1.892803 -2.64 0.008 -8.709825 -1.290175

LD3 vs LD1 | -7.5 1.892803 -3.96 0.000 -11.20982 -3.790175

LD4 vs LD1 | -2.333333 1.892803 -1.23 0.218 -6.043158 1.376492

LD3 vs LD2 | -2.5 1.892803 -1.32 0.187 -6.209825 1.209825

LD4 vs LD2 | 2.666667 1.892803 1.41 0.159 -1.043158 6.376492

LD4 vs LD3 | 5.166667 1.892803 2.73 0.006 1.456842 8.876492

---------------------------------------------------------------------------------

**Table S6: A mixed-effects linear regression model for pupation of *w*AlbB strain under the four diets.**

| **Effect type** | **Source** | **Estimate** | **95% CI** | **p-value** |
| --- | --- | --- | --- | --- |
| Fixed | Intercept | 98.17 | 95.24, 101.09 | <0.001 |
|  | LD2 | -7.17 | -11.31, -3.03 | 0.001 |
|  | LD3 | -9.67 | -13.81, -5.53 | <0.001 |
|  | LD4 | -1.33 | -5.47, 2.81 | 0.528 |
| Random | Generation (Var) | 1.89e-18 | 8.62e-42, 414079.50 | - |
|  | Replicate within generation (Var) | 1.08e-18 | 6.90e-34, 0.0017 | - |
| Residual variance |  | 13.38 | 7.60, 23.56 | - |
| Model fit | AIC =144.36, BIC =152.61 | - | - | - |
| ICC | Generation=1.41e-19, Replicate=2.22e-19 | - | - | - |
| R2 (marginal) | 0.55 | - | - | - |
| R2 (Conditional) | 0.55 | - | - | - |
| Wald chi-square, P-value | 28.73, P<0.001 |  |  |  |
| LR test, P-value | 5.7e-14, P = 1.000 |  |  |  |

Pairwise comparisons of marginal linear predictions

Margins : asbalanced

---------------------------------------------------------------------------------

| Unadjusted Unadjusted

| Contrast Std. Err. z P>|z| [95% Conf. Interval]

----------------+----------------------------------------------------------------

noofpupalformed |

diet1 |

LD2 vs LD1 | -7.166667 2.112025 -3.39 0.001 -11.30616 -3.027174

LD3 vs LD1 | -9.666667 2.112025 -4.58 0.000 -13.80616 -5.527174

LD4 vs LD1 | -1.333333 2.112025 -0.63 0.528 -5.472826 2.806159

LD3 vs LD2 | -2.5 2.112025 -1.18 0.237 -6.639492 1.639492

LD4 vs LD2 | 5.833333 2.112025 2.76 0.006 1.693841 9.972826

LD4 vs LD3 | 8.333333 2.112025 3.95 0.000 4.193841 12.47283

---------------------------------------------------------------------------------

**Table S7: A mixed-effects linear regression model for pupation of *w*Mel strain under the four diets.**

| **Effect type** | **Source** | **Estimate** | **95% CI** | **p-value** |
| --- | --- | --- | --- | --- |
| Fixed | Intercept | 96.67 | 92.18, 101.15 | <0.001 |
|  | LD2 | -8.50 | -14.84, -2.16 | 0.009 |
|  | LD3 | -1.33 | -7.67, 5.01 | 0.680 |
|  | LD4 | 1.33 | -5.01, 7.67 | 0.680 |
| Random | Generation (Var) | 2.87e-13 | 3.00e-44, 2.75e+18 | - |
|  | Replicate within generation (Var) | 1.17e-18 | 2.86e-35, 0.048 | - |
| Residual variance |  | 31.40 | 17.83, 55.28 | - |
| Model fit | AIC =164.83, BIC =173.08 | - | - | - |
| ICC | Generation=9.15e-15, Replicate=9.15e-15 | - | - | - |
| R2 (marginal) | 0.32 | - | - | - |
| R2 (Conditional) | 0.32 | - | - | - |
| Wald chi-square, P-value | 11.04, P=0.011 |  |  |  |
| LR test, P-value | 2.8e-14, P = 1.000 |  |  |  |

Pairwise comparisons of marginal linear predictions

Margins : asbalanced

---------------------------------------------------------------------------------

| Unadjusted Unadjusted

| Contrast Std. Err. z P>|z| [95% Conf. Interval]

----------------+----------------------------------------------------------------

noofpupalformed |

diet1 |

LD2 vs LD1 | -8.5 3.235008 -2.63 0.009 -14.8405 -2.159501

LD3 vs LD1 | -1.333333 3.235008 -0.41 0.680 -7.673833 5.007166

LD4 vs LD1 | 1.333333 3.235008 0.41 0.680 -5.007166 7.673833

LD3 vs LD2 | 7.166667 3.235008 2.22 0.027 .8261672 13.50717

LD4 vs LD2 | 9.833333 3.235008 3.04 0.002 3.492834 16.17383

LD4 vs LD3 | 2.666667 3.235008 0.82 0.410 -3.673833 9.007166

---------------------------------------------------------------------------------

**Table S8: A mixed-effects linear regression model for adult emergence of uninfected *Ae. aegypti* under the four diets.**

| **Effect type** | **Source** | **Estimate** | **95% CI** | **p-value** |
| --- | --- | --- | --- | --- |
| Fixed | Intercept | 97.17 | 92.90, 101.43 | <0.001 |
|  | LD2 | -4.00 | -7.63, -0.37 | 0.031 |
|  | LD3 | -6.50 | -10.13, -2.87 | <0.001 |
|  | LD4 | -1.33 | -4.96, 2.30 | 0.472 |
| Random | Generation (Var) | 6.03 | 0.64, 56.68 | - |
|  | Replicate within generation (Var) | 4.00e-21 | 5.20e-36, 3.08e-06 | - |
| Residual variance |  | 10.29 | 5.70, 18.59 | - |
| Model fit | AIC =142.23, BIC =150.48 | - | - | - |
| ICC | Generation=0.37, Replicate=0.37 | - | - | - |
| R2 (marginal) | 0.29 | - | - | - |
| R2 (Conditional) | 0.55 | - | - | - |
| Wald chi-square, P-value | 14.58, P=0.002 |  |  |  |
| LR test, P-value | 6.90, P = 0.032 |  |  |  |

Pairwise comparisons of marginal linear predictions

Margins : asbalanced

----------------------------------------------------------------------------------

| Unadjusted Unadjusted

| Contrast Std. Err. z P>|z| [95% Conf. Interval]

-----------------+----------------------------------------------------------------

noofadultemerged |

diet1 |

LD2 vs LD1 | -4 1.852346 -2.16 0.031 -7.630532 -.369468

LD3 vs LD1 | -6.5 1.852346 -3.51 0.000 -10.13053 -2.869468

LD4 vs LD1 | -1.333333 1.852346 -0.72 0.472 -4.963865 2.297199

LD3 vs LD2 | -2.5 1.852346 -1.35 0.177 -6.130532 1.130532

LD4 vs LD2 | 2.666667 1.852346 1.44 0.150 -.9638653 6.297199

LD4 vs LD3 | 5.166667 1.852346 2.79 0.005 1.536135 8.797199

----------------------------------------------------------------------------------

**Table S9: A mixed-effects linear regression model for adult emergence of *w*AlbB strain under the four diets.**

| **Effect type** | **Source** | **Estimate** | **95% CI** | **p-value** |
| --- | --- | --- | --- | --- |
| Fixed | Intercept | 97.67 | 94.87, 100.46 | <0.001 |
|  | LD2 | -7.00 | -10.95, -3.05 | 0.001 |
|  | LD3 | -9.33 | -13.29, -5.38 | <0.001 |
|  | LD4 | -0.83 | -4.79, 3.12 | 0.679 |
| Random | Generation (Var) | 1.42e-19 | 0.00, Not estimable | - |
|  | Replicate within generation (Var) | 3.03e-20 | 1.26e-33, 7.29e-07 | - |
| Residual variance |  | 12.20 | 6.93, 21.48 | - |
| Model fit | AIC =142.15, BIC =150.39 | - | - | - |
| ICC | Generation=1.17e-20, Replicate=1.41e-20 | - | - | - |
| R2 (marginal) | 0.57 | - | - | - |
| R2 (Conditional) | 0.57 | - | - | - |
| Wald chi-square, P-value | 31.04, P<0.001 |  |  |  |
| LR test, P-value | 2.8e-14, P = 1.00 |  |  |  |

Pairwise comparisons of marginal linear predictions

Margins : asbalanced

----------------------------------------------------------------------------------

| Unadjusted Unadjusted

| Contrast Std. Err. z P>|z| [95% Conf. Interval]

-----------------+----------------------------------------------------------------

noofadultemerged |

diet1 |

LD2 vs LD1 | -7 2.016713 -3.47 0.001 -10.95268 -3.047316

LD3 vs LD1 | -9.333333 2.016713 -4.63 0.000 -13.28602 -5.380649

LD4 vs LD1 | -.8333333 2.016713 -0.41 0.679 -4.786017 3.119351

LD3 vs LD2 | -2.333333 2.016713 -1.16 0.247 -6.286017 1.619351

LD4 vs LD2 | 6.166667 2.016713 3.06 0.002 2.213983 10.11935

LD4 vs LD3 | 8.5 2.016713 4.21 0.000 4.547316 12.45268

----------------------------------------------------------------------------------

**Table S10: A mixed-effects linear regression model for adult emergence of *w*Mel strain under the four diets.**

| **Effect type** | **Source** | **Estimate** | **95% CI** | **p-value** |
| --- | --- | --- | --- | --- |
| Fixed | Intercept | 95.50 | 90.70, 100.30 | <0.001 |
|  | LD2 | -8.00 | -14.03, -1.97 | 0.009 |
|  | LD3 | -0.17 | -6.20, 5.86 | 0.957 |
|  | LD4 | 4.26e-14 | -6.03, 6.03 | 1.000 |
| Random | Generation (Var) | 2.51 | 0.04, 139.18 | - |
|  | Replicate within generation (Var) | 8.40e-14 | 6.76e-31, 10424.98 | - |
| Residual variance |  | 28.40 | 15.09, 53.44 | - |
| Model fit | AIC =163.87, BIC =172.11 | - | - | - |
| ICC | Generation=0.08, Replicate=0.08 | - | - | - |
| R2 (marginal) | 0.28 | - | - | - |
| R2 (Conditional) | 0.34 | - | - | - |
| Wald chi-square, P-value | 10.00, P=0.018 |  |  |  |
| LR test, P-value | 0.59, P = 0.746 |  |  |  |

Pairwise comparisons of marginal linear predictions

Margins : asbalanced

----------------------------------------------------------------------------------

| Unadjusted Unadjusted

| Contrast Std. Err. z P>|z| [95% Conf. Interval]

-----------------+----------------------------------------------------------------

noofadultemerged |

diet1 |

LD2 vs LD1 | -8 3.076774 -2.60 0.009 -14.03037 -1.969633

LD3 vs LD1 | -.1666667 3.076774 -0.05 0.957 -6.197033 5.8637

LD4 vs LD1 | 4.26e-14 3.076774 0.00 1.000 -6.030367 6.030367

LD3 vs LD2 | 7.833333 3.076774 2.55 0.011 1.802967 13.8637

LD4 vs LD2 | 8 3.076774 2.60 0.009 1.969633 14.03037

LD4 vs LD3 | .1666667 3.076774 0.05 0.957 -5.8637 6.197033

----------------------------------------------------------------------------------

**Table S11: A mixed-effects linear regression model for fecundity of uninfected *Ae. aegypti***

**under the four diets.**

| **Effect type** | **Source** | **Estimate** | **95% CI** | **p-value** |
| --- | --- | --- | --- | --- |
| Fixed | Intercept | 64.74 | 45.24, 84.24 | <0.001 |
|  | LD2 | 8.46 | -13.32, 30.25 | 0.446 |
|  | LD3 | -25.50 | -47.29, -3.72 | 0.022 |
|  | LD4 | 15.81 | -5.97, 37.60 | 0.155 |
| Random | Generation (Var) | 74.46 | 4.60, 1204.70 | - |
|  | Replicate within generation (Var) | 2.83e-11 | 0, Not estimable | - |
| Residual variance |  | 370.61 | 205.25, 669.22 | - |
| Model fit | AIC =226.53, BIC =234.77 | - | - | - |
| ICC | Generation=0.17, Replicate=0.17 | - | - | - |
| R2 (marginal) | 0.36 | - | - | - |
| R2 (Conditional) | 0.47 | - | - | - |
| Wald chi-square, P-value | 15.73, P=0.001 |  |  |  |
| LR test, P-value | 1.94, P = 0.379 |  |  |  |

Pairwise comparisons of marginal linear predictions

Margins : asbalanced

------------------------------------------------------------------------------

| Unadjusted Unadjusted

| Contrast Std. Err. z P>|z| [95% Conf. Interval]

-------------+----------------------------------------------------------------

fecundity |

diet1 |

LD2 vs LD1 | 8.461667 11.11476 0.76 0.446 -13.32286 30.2462

LD3 vs LD1 | -25.505 11.11476 -2.29 0.022 -47.28953 -3.720469

LD4 vs LD1 | 15.81167 11.11476 1.42 0.155 -5.972861 37.5962

LD3 vs LD2 | -33.96667 11.11476 -3.06 0.002 -55.7512 -12.18214

LD4 vs LD2 | 7.350002 11.11476 0.66 0.508 -14.43453 29.13453

LD4 vs LD3 | 41.31667 11.11476 3.72 0.000 19.53214 63.1012

------------------------------------------------------------------------------

**Table S12: A mixed-effects linear regression model for fecundity of *w*AlbB strain under the four diets.**

| **Effect type** | **Source** | **Estimate** | **95% CI** | **p-value** |
| --- | --- | --- | --- | --- |
| Fixed | Intercept | 53.51 | 40.02, 67.00 | <0.001 |
|  | LD2 | -1.14 | -19.57, 17.28 | 0.903 |
|  | LD3 | -9.23 | -27.65, 9.19 | 0.326 |
|  | LD4 | 3.44 | -14.98, 21.86 | 0.715 |
| Random | Generation (Var) | 1.06e-08 | 8.48e-34, 1.33e+17 | - |
|  | Replicate within generation (Var) | 19.32 | 0.08, 4685.55 | - |
| Residual variance |  | 265.05 | 137.91, 509.40 | - |
| Model fit | AIC =217.56, BIC =225.81 | - | - | - |
| ICC | Generation=3.74e-11, Replicate=0.07 | - | - | - |
| R2 (marginal) | 0.07 | - | - | - |
| R2 (Conditional) | 0.14 | - | - | - |
| Wald chi-square, P-value | 1.95, P=0.582 |  |  |  |
| LR test, P-value | 0.15, P = 0.926 |  |  |  |

Pairwise comparisons of marginal linear predictions

Margins : asbalanced

------------------------------------------------------------------------------

| Unadjusted Unadjusted

| Contrast Std. Err. z P>|z| [95% Conf. Interval]

-------------+----------------------------------------------------------------

fecundity |

diet1 |

LD2 vs LD1 | -1.145 9.399423 -0.12 0.903 -19.56753 17.27753

LD3 vs LD1 | -9.228333 9.399423 -0.98 0.326 -27.65086 9.194197

LD4 vs LD1 | 3.438334 9.399423 0.37 0.715 -14.9842 21.86086

LD3 vs LD2 | -8.083334 9.399423 -0.86 0.390 -26.50586 10.3392

LD4 vs LD2 | 4.583333 9.399423 0.49 0.626 -13.8392 23.00586

LD4 vs LD3 | 12.66667 9.399423 1.35 0.178 -5.755863 31.0892

------------------------------------------------------------------------------

**Table S13: A mixed-effects linear regression model for fecundity of *w*Mel strain under the four diets.**

| **Effect type** | **Source** | **Estimate** | **95% CI** | **p-value** |
| --- | --- | --- | --- | --- |
| Fixed | Intercept | 58.42 | 32.82, 84.03 | <0.001 |
|  | LD2 | 0.21 | -13.29, 13.71 | 0.976 |
|  | LD3 | -9.06 | -22.56, 4.44 | 0.189 |
|  | LD4 | -7.17 | -20.68, 6.33 | 0.298 |
| Random | Generation (Var) | 293.88 | 38.24, 2258.34 | - |
|  | Replicate within generation (Var) | 3.63e-18 | 0, Not estimable | - |
| Residual variance |  | 142.36 | 78.84, 257.06 | - |
| Model fit | AIC =207.61, BIC =215.85 | - | - | - |
| ICC | Generation=0.67, Replicate=0.57 | - | - | - |
| R2 (marginal) | 0.04 | - | - | - |
| R2 (Conditional) | 0.69 | - | - | - |
| Wald chi-square, P-value | 2.92, P=0.404 |  |  |  |
| LR test, P-value | 20.38, P <0.001 |  |  |  |

Pairwise comparisons of marginal linear predictions

Margins : asbalanced

------------------------------------------------------------------------------

| Unadjusted Unadjusted

| Contrast Std. Err. z P>|z| [95% Conf. Interval]

-------------+----------------------------------------------------------------

fecundity |

diet1 |

LD2 vs LD1 | .2083321 6.888581 0.03 0.976 -13.29304 13.7097

LD3 vs LD1 | -9.058334 6.888581 -1.31 0.189 -22.5597 4.443036

LD4 vs LD1 | -7.175 6.888581 -1.04 0.298 -20.67637 6.32637

LD3 vs LD2 | -9.266666 6.888581 -1.35 0.179 -22.76804 4.234704

LD4 vs LD2 | -7.383332 6.888581 -1.07 0.284 -20.8847 6.118038

LD4 vs LD3 | 1.883334 6.888581 0.27 0.785 -11.61804 15.3847

------------------------------------------------------------------------------

**Table S14: A mixed-effects linear regression model for male-female ratio of uninfected *Ae. aegypti* under four diets.**

| **Effect type** | **Source** | **Estimate** | **95% CI** | **p-value** |
| --- | --- | --- | --- | --- |
| Fixed | Intercept | 49.23 | 48.36, 50.10 | <0.001 |
|  | LD2 | -1.09 | -2.32, 0.14 | 0.084 |
|  | LD3 | -0.88 | -2.11, 0.35 | 0.160 |
|  | LD4 | -0.70 | -1.93, 0.53 | 0.262 |
| Random | Generation (Var) | 3.75e-26 | 9.98e-50, 0.01 | - |
|  | Replicate within generation (Var) | 2.19e-21 | 9.19e-35, 5.20e-08 | - |
| Residual variance |  | 1.18 | 0.67, 2.08 | - |
| Model fit | AIC =86.12, BIC =94.37 | - | - | - |
| ICC | Generation=3.18e-26, Replicate=1.85e-21 | - | - | - |
| R2 (marginal) | 0.13 | - | - | - |
| R2 (Conditional) | 0.13 | - | - | - |
| Wald chi-square, P-value | 3.39, P=0.335 |  |  |  |
| LR test, P-value | 0.00, P = 1.00 |  |  |  |

Pairwise comparisons of marginal linear predictions

Margins : asbalanced

------------------------------------------------------------------------------

| Unadjusted Unadjusted

| Contrast Std. Err. z P>|z| [95% Conf. Interval]

-------------+----------------------------------------------------------------

female_per |

diet1 |

LD2 vs LD1 | -1.086288 .6276943 -1.73 0.084 -2.316547 .1439697

LD3 vs LD1 | -.8811423 .6276943 -1.40 0.160 -2.1114 .3491159

LD4 vs LD1 | -.7041721 .6276943 -1.12 0.262 -1.93443 .5260861

LD3 vs LD2 | .2051462 .6276943 0.33 0.744 -1.025112 1.435404

LD4 vs LD2 | .3821163 .6276943 0.61 0.543 -.8481419 1.612375

LD4 vs LD3 | .1769702 .6276943 0.28 0.778 -1.053288 1.407228

------------------------------------------------------------------------------

**Table S15: A mixed-effects linear regression model for male-female ratio of *w*AlbB strain under four diets.**

| **Effect type** | **Source** | **Estimate** | **95% CI** | **p-value** |
| --- | --- | --- | --- | --- |
| Fixed | Intercept | 48.42 | 46.89, 50.06 | <0.001 |
|  | LD2 | -0.67 | -2.71, 1.36 | 0.517 |
|  | LD3 | -2.09 | -4.12, -0.05 | 0.045 |
|  | LD4 | -1.82 | -3.85, 0.22 | 0.081 |
| Random | Generation (Var) | 0.23 | 0.003, 17.33 | - |
|  | Replicate within generation (Var) | 3.10e-20 | 8.96e-36, 0.000107 | - |
| Residual variance |  | 3.24 | 1.80, 5.86 | - |
| Model fit | AIC =111.59, BIC =119.83 | - | - | - |
| ICC | Generation=0.07, Replicate=0.07 | - | - | - |
| R2 (marginal) | 0.18 | - | - | - |
| R2 (Conditional) | 0.23 | - | - | - |
| Wald chi-square, P-value | 5.31, P=0.151 |  |  |  |
| LR test, P-value | 0.41, P = 0.814 |  |  |  |

Pairwise comparisons of marginal linear predictions

Margins : asbalanced

------------------------------------------------------------------------------

| Unadjusted Unadjusted

| Contrast Std. Err. z P>|z| [95% Conf. Interval]

-------------+----------------------------------------------------------------

female_per |

diet1 |

LD2 vs LD1 | -.6735414 1.039998 -0.65 0.517 -2.711901 1.364818

LD3 vs LD1 | -2.086294 1.039998 -2.01 0.045 -4.124653 -.0479349

LD4 vs LD1 | -1.816618 1.039998 -1.75 0.081 -3.854977 .2217413

LD3 vs LD2 | -1.412753 1.039998 -1.36 0.174 -3.451112 .6256065

LD4 vs LD2 | -1.143077 1.039998 -1.10 0.272 -3.181436 .8952827

LD4 vs LD3 | .2696762 1.039998 0.26 0.795 -1.768683 2.308035

------------------------------------------------------------------------------

**Table S16: A mixed-effects linear regression model for male-female ratio of *w*Mel strain under four diets.**

| **Effect type** | **Source** | **Estimate** | **95% CI** | **p-value** |
| --- | --- | --- | --- | --- |
| Fixed | Intercept | 48.70 | 47.79, 49.62 | <0.001 |
|  | LD2 | -0.46 | -1.43, 0.51 | 0.356 |
|  | LD3 | -0.28 | -1.25, 0.69 | 0.573 |
|  | LD4 | -0.50 | -1.47, 0.47 | 0.310 |
| Random | Generation (Var) | 4.66e-24 | Not estimable | - |
|  | Replicate within generation (Var) | 0.57 | 0.13, 2.59 | - |
| Residual variance |  | 0.74 | 0.38, 1.42 | - |
| Model fit | AIC =81.25, BIC =88.31 | - | - | - |
| ICC | Generation=3.56e-24, Replicate=0.44 | - | - | - |
| R2 (marginal) | 0.03 | - | - | - |
| R2 (Conditional) | 0.45 | - | - | - |
| Wald chi-square, P-value | 1.27, P=0.74 |  |  |  |
| LR test, P-value | 5.30, P = 0.071 |  |  |  |

Pairwise comparisons of marginal linear predictions

Margins : asbalanced

------------------------------------------------------------------------------

| Unadjusted Unadjusted

| Contrast Std. Err. z P>|z| [95% Conf. Interval]

-------------+----------------------------------------------------------------

female_per |

diet1 |

LD2 vs LD1 | -.4571374 .4956398 -0.92 0.356 -1.428574 .5142987

LD3 vs LD1 | -.2791716 .4956398 -0.56 0.573 -1.250608 .6922645

LD4 vs LD1 | -.5033067 .4956398 -1.02 0.310 -1.474743 .4681294

LD3 vs LD2 | .1779658 .4956398 0.36 0.720 -.7934703 1.149402

LD4 vs LD2 | -.0461693 .4956398 -0.09 0.926 -1.017605 .9252668

LD4 vs LD3 | -.2241351 .4956398 -0.45 0.651 -1.195571 .747301

------------------------------------------------------------------------------
