# Supplementary material for: Life table variations in Wolbachia-transinfected (wMel & wAlbB strains) and uninfected Aedes aegypti: the role of various larval diets
Source: Front Insect Sci. 2025 Dec 12;5:1679816. doi: 10.3389/finsc.2025.1679816 (PMC12741118; doi:10.3389/finsc.2025.1679816)
Supplement: Supplementary File 2 — Raw data of mosquito survival in each stage under different diets (F0 & F1 generation). [file Table3.docx]

**Table S17. Raw data of mosquito survival in each stage under different diets (F0 generation)**

| **Diets** | **Mosquito strain** | **Replicates** | **First Instar larvae** | **First-second instar** | **second -third Instar** | **Third -fourth Instar** | **fourth -pupae** | **Pupae-adult** | **Emergence** | |
| --- | --- | --- | --- | --- | --- | --- | --- | --- | --- | --- |
|  |  |  |  |  |  |  |  |  | **Male** | **Female** |
| **LD1** | **Uninfected** | I | 100 | 100 | 100 | 99 | 99 | 99 | 50 | 49 |
|  |  | II | 100 | 99 | 98 | 98 | 98 | 98 | 49 | 49 |
|  |  | III | 100 | 100 | 100 | 100 | 100 | 100 | 51 | 49 |
|  | ***w*ALB** | I | 100 | 100 | 100 | 100 | 99 | 99 | 50 | 49 |
|  |  | II | 100 | 100 | 99 | 98 | 97 | 97 | 49 | 48 |
|  |  | III | 100 | 100 | 100 | 97 | 97 | 96 | 48 | 48 |
|  | ***w*Mel** | I | 100 | 100 | 97 | 96 | 93 | 93 | 47 | 46 |
|  |  | II | 100 | 100 | 100 | 98 | 97 | 97 | 50 | 47 |
|  |  | III | 100 | 100 | 100 | 97 | 95 | 94 | 48 | 46 |
| **LD2** | **Uninfected** | I | 100 | 100 | 100 | 100 | 100 | 100 | 55 | 45 |
|  |  | II | 100 | 100 | 99 | 98 | 98 | 98 | 48 | 50 |
|  |  | III | 100 | 100 | 100 | 97 | 97 | 91 | 48 | 43 |
|  | ***w*ALB** | I | 100 | 100 | 99 | 98 | 98 | 98 | 52 | 44 |
|  |  | II | 100 | 99 | 97 | 97 | 97 | 96 | 50 | 46 |
|  |  | III | 100 | 100 | 98 | 98 | 98 | 97 | 49 | 48 |
|  | ***w*Mel** | I | 100 | 99 | 99 | 99 | 99 | 99 | 50 | 49 |
|  |  | II | 100 | 98 | 98 | 98 | 98 | 98 | 49 | 47 |
|  |  | III | 100 | 100 | 98 | 98 | 98 | 98 | 49 | 47 |
| **LD3** | **Uninfected** | I | 100 | 100 | 96 | 96 | 96 | 96 | 49 | 47 |
|  |  | II | 100 | 100 | 97 | 97 | 97 | 97 | 50 | 47 |
|  |  | III | 100 | 96 | 96 | 96 | 96 | 96 | 50 | 46 |
|  | ***w*ALB** | I | 100 | 96 | 96 | 96 | 96 | 88 | 45 | 42 |
|  |  | II | 100 | 100 | 100 | 100 | 86 | 86 | 44 | 42 |
|  |  | III | 100 | 97 | 97 | 95 | 88 | 81 |  |  |
|  | ***w*Mel** | I | 100 | 100 | 100 | 100 | 100 | 96 | 49 | 47 |
|  |  | II | 100 | 100 | 98 | 98 | 98 | 92 | 47 | 45 |
|  |  | III | 100 | 100 | 100 | 100 | 95 | 95 | 50 | 45 |
| **LD4** | **Uninfected** | I | 100 | 99 | 99 | 99 | 99 | 99 | 50 | 49 |
|  |  | II | 100 | 100 | 99 | 98 | 93 | 91 | 47 | 44 |
|  |  | III | 100 | 100 | 99 | 97 | 97 | 97 | 50 | 47 |
|  | ***w*ALB** | I | 100 | 100 | 100 | 98 | 97 | 97 | 50 | 47 |
|  |  | II | 100 | 100 | 98 | 96 | 95 | 95 | 49 | 46 |
|  |  | III | 100 | 100 | 98 | 98 | 96 | 96 | 50 | 46 |
|  | ***w*Mel** | I | 100 | 100 | 98 | 97 | 95 | 95 | 49 | 46 |
|  |  | II | 100 | 100 | 100 | 97 | 97 | 96 | 49 | 47 |
|  |  | III | 100 | 99 | 99 | 99 | 99 | 99 | 51 | 48 |

**Table S18. Raw data of mosquito survival in each stage under different diet (F1 generation)**

| **F0 Generation** | **Mosquito strain** | **Replicates** | **First Instar larvae** | **First-second instar** | **second -third Instar** | **Third -fourth Instar** | **fourth -pupae** | **Pupae-adult** | **Emergence** | |
| --- | --- | --- | --- | --- | --- | --- | --- | --- | --- | --- |
|  |  |  |  |  |  |  |  |  | **Male** | **Female** |
| **LD1** | **Uninfected** | I | 100 | 100 | 100 | 99 | 98 | 97 | 47 | 46 |
|  |  | II | 100 | 100 | 100 | 100 | 98 | 96 | 50 | 46 |
|  |  | III | 100 | 100 | 100 | 100 | 100 | 99 | 49 | 48 |
|  | ***w*ALB** | I | 100 | 100 | 100 | 98 | 98 | 98 | 50 | 47 |
|  |  | II | 100 | 100 | 100 | 100 | 100 | 100 | 50 | 48 |
|  |  | III | 100 | 100 | 100 | 100 | 99 | 99 | 55 | 44 |
|  | ***w*Mel** | I | 100 | 100 | 100 | 99 | 99 | 98 | 49 | 47 |
|  |  | II | 100 | 100 | 100 | 100 | 99 | 99 | 48 | 46 |
|  |  | III | 100 | 100 | 100 | 100 | 100 | 99 | 52 | 47 |
| **LD2** | **Uninfected** | I | 100 | 100 | 100 | 100 | 99 | 89 | 45 | 44 |
|  |  | II | 100 | 100 | 100 | 99 | 94 | 91 | 47 | 44 |
|  |  | III | 100 | 100 | 100 | 97 | 95 | 90 | 47 | 43 |
|  | ***w*ALB** | I | 100 | 100 | 100 | 100 | 100 | 86 | 45 | 41 |
|  |  | II | 100 | 100 | 100 | 100 | 97 | 82 | 42 | 40 |
|  |  | III | 100 | 100 | 100 | 97 | 95 | 87 | 46 | 41 |
|  | ***w*Mel** | I | 100 | 100 | 100 | 94 | 93 | 84 | 43 | 41 |
|  |  | II | 100 | 100 | 96 | 84 | 83 | 77 | 40 | 37 |
|  |  | III | 100 | 100 | 90 | 87 | 86 | 73 | 40 | 33 |
| **LD3** | **Uninfected** | I | 100 | 100 | 100 | 99 | 90 | 88 | 46 | 42 |
|  |  | II | 100 | 100 | 100 | 100 | 91 | 85 | 44 | 41 |
|  |  | III | 100 | 100 | 100 | 99 | 87 | 82 | 42 | 40 |
|  | ***w*ALB** | I | 100 | 100 | 100 | 100 | 98 | 86 | 47 | 39 |
|  |  | II | 100 | 100 | 100 | 98 | 95 | 92 | 50 | 42 |
|  |  | III | 100 | 100 | 98 | 98 | 98 | 94 | 49 | 45 |
|  | ***w*Mel** | I | 100 | 100 | 100 | 100 | 98 | 97 | 49 | 48 |
|  |  | II | 100 | 100 | 100 | 97 | 97 | 95 | 50 | 45 |
|  |  | III | 100 | 100 | 100 | 100 | 100 | 97 | 50 | 47 |
| **LD4** | **Uninfected** | I | 100 | 100 | 100 | 99 | 96 | 94 | 48 | 46 |
|  |  | II | 100 | 100 | 99 | 98 | 97 | 96 | 49 | 47 |
|  |  | III | 100 | 100 | 99 | 99 | 98 | 98 | 52 | 46 |
|  | ***w*ALB** | I | 100 | 100 | 100 | 98 | 98 | 98 | 55 | 44 |
|  |  | II | 100 | 100 | 100 | 100 | 100 | 99 | 51 | 46 |
|  |  | III | 100 | 100 | 100 | 100 | 100 | 100 | 55 | 42 |
|  | ***w*Mel** | I | 100 | 100 | 100 | 99 | 99 | 99 | 45 | 43 |
|  |  | II | 100 | 100 | 100 | 100 | 99 | 99 | 48 | 47 |
|  |  | III | 100 | 100 | 100 | 100 | 100 | 100 | 55 | 45 |

**Table 19. Fecundity of *Wolbachia*-transinfected *w*AlbB (Pud) and *w*Mel (Pud) and uninfected *Ae. aegypti* lines under different diets in F0 and F1 generations.**

| **Larval diets** | ***Ae. aegypti* lines** | **Replicates** | | **No: of females alive at Oviposition** | | **Total no. of eggs laid** | | **Fecundity (%)** | |
| --- | --- | --- | --- | --- | --- | --- | --- | --- | --- |
| **LD1** |  | **F0** | **F1** | **F0** | **F1** | **F0** | **F1** | **F0** | **F1** |
|  | **uninfected** | 1 | 1 | 35 | 46 | 2701 | 2531 | 77.17 | 55.0 |
|  |  | 2 | 2 | 25 | 47 | 2155 | 2145 | 86.2 | 45.6 |
|  |  | 3 | 3 | 30 | 45 | 2651 | 1623 | 88.36 | 36.1 |
|  | ***w*AlbB (Pud)** | 1 | 1 | 32 | 48 | 1450 | 4063 | 45.31 | 84.6 |
|  |  | 2 | 2 | 19 | 47 | 1087 | 2442 | 57.21 | 52.0 |
|  |  | 3 | 3 | 31 | 46 | 1186 | 2011 | 38.25 | 43.7 |
|  | ***w*Mel (Pud)** | 1 | 1 | 18 | 46 | 1302 | 2733 | 72.33 | 59.4 |
|  |  | 2 | 2 | 30 | 46 | 2388 | 1492 | 79.6 | 32.4 |
|  |  | 3 | 3 | 38 | 47 | 2334 | 2133 | 61.42 | 45.4 |
| **LD2** | **Uninfected** | **F0** | **F1** | **F0** | **F1** | **F0** | **F1** | **F0** | **F1** |
|  |  | 1 | 1 | 30 | 46 | 2569 | 3274 | 85.6 | 71.2 |
|  |  | 2 | 2 | 35 | 47 | 1962 | 3913 | 56.1 | 83.3 |
|  |  | 3 | 3 | 27 | 45 | 1954 | 3178 | 72.4 | 70.6 |
|  | ***w*AlbB (Pud)** | 1 | 1 | 28 | 47 | 1240 | 2195 | 44.3 | 46.7 |
|  |  | 2 | 2 | 18 | 47 | 1682 | 2015 | 93.4 | 42.9 |
|  |  | 3 | 3 | 26 | 46 | 1238 | 1807 | 47.6 | 39.3 |
|  | ***w*Mel (Pud)** | 1 | 1 | 18 | 46 | 1375 | 2097 | 76.4 | 45.6 |
|  |  | 2 | 2 | 23 | 46 | 1057 | 2626 | 46.0 | 57.1 |
|  |  | 3 | 3 | 18 | 48 | 1351 | 2476 | 75.1 | 51.6 |
| **LD3** |  | **F0** | **F1** | **F0** | **F1** | **F0** | **F1** | **F0** | **F1** |
|  | **Uninfected** | 1 | 1 | 12 | 47 | 630 | 595 | 52.5 | 12.7 |
|  |  | 2 | 2 | 7 | 45 | 412 | 304 | 58.5 | 6.8 |
|  |  | 3 | 3 | 6 | 48 | 573 | 451 | 95.5 | 9.4 |
|  | ***w*AlbB (Pud)** | 1 | 1 | 25 | 47 | 1111 | 2564 | 44.4 | 54.6 |
|  |  | 2 | 2 | 19 | 46 | 430 | 2201 | 22.6 | 47.8 |
|  |  | 3 | 3 | 18 | 47 | 696 | 2706 | 38.7 | 57.6 |
|  | ***w*Mel (Pud)** | 1 | 1 | 32 | 48 | 2079 | 1321 | 65.0 | 27.5 |
|  |  | 2 | 2 | 21 | 47 | 1589 | 1323 | 75.7 | 28.1 |
|  |  | 3 | 3 | 12 | 46 | 929 | 1033 | 77.4 | 22.5 |
| **LD4** |  | **F0** | **F1** | **F0** | **F1** | **F0** | **F1** | **F0** | **F1** |
|  | **Uninfected** | 1 | 1 | 26 | 47 | 2321 | 3964 | 89.3 | 84.3 |
|  |  | 2 | 2 | 25 | 46 | 1193 | 3998 | 47.7 | 86.9 |
|  |  | 3 | 3 | 28 | 47 | 2434 | 4140 | 87.0 | 88.1 |
|  | ***w*AlbB (Pud)** | 1 | 1 | 20 | 46 | 667 | 2903 | 33.4 | 63.1 |
|  |  | 2 | 2 | 25 | 46 | 1708 | 3712 | 68.3 | 80.7 |
|  |  | 3 | 3 | 20 | 45 | 484 | 3240 | 24.2 | 72.0 |
|  | ***w*Mel (Pud)** | 1 | 1 | 26 | 47 | 2142 | 928 | 82.4 | 19.7 |
|  |  | 2 | 2 | 31 | 46 | 2460 | 1756 | 79.4 | 38.2 |
|  |  | 3 | 3 | 37 | 48 | 1947 | 753 | 72.1 | 15.7 |
